# Neural control meets biomechanics in the motor assessment of neurological disorders: a narrative review

**DOI:** 10.3389/fncir.2025.1608328

**Published:** 2025-06-27

**Authors:** Mirjam Bonanno, Paolo De Pasquale, Bartolo Fonti, Elvira Gjonaj, Simona De Salvo, Angelo Quartarone, Rocco Salvatore Calabrò

**Affiliations:** ^1^IRCCS Centro Neurolesi Bonino-Pulejo, Messina, Italy; ^2^Department of Biomedical, Dental Sciences and Morphological and Functional Images, University of Messina, Messina, Italy

**Keywords:** neurobiomechanical assessment, cortico-muscular coherence, cortico-spinal pathway, neurological disorders, neurorehabilitation, neurophysiology, biomechanics, computational approaches

## Abstract

The emerging concept of “neurobiomechanics” embodies an integrative approach, bringing together insights from functional anatomy, the physiology of the musculoskeletal and central nervous systems, physics, and computer science. By examining human movement under normal, optimal, and pathological conditions, neurobiomechanics aims to unravel the intricate mechanisms driving motor function and dysfunction, offering a comprehensive perspective on disorders such as acquired brain injury and neurodegenerative diseases. In this narrative review, we sought to explore the “neurobiomechanics” as a potential approach to investigate both neural and biomechanical aspects of human motion, trying to answer the following questions: (1) “Which technologies can perform a neurobiomechanical assessment in neurological patients?,” (2) “What are the key neurophysiological and biomechanical parameters?,” (3) “How can we translate this approach from research to clinical practice?.” We have found that, to assess/understand a patient’s dysfunctional patterns, it is necessary to evaluate both neurophysiology and biomechanics in a complementary manner. In other words, assessing one aspect without the other is not sufficient, as this may lead to incomplete evaluations from both a functional and methodological perspective.

## 1 Introduction

Human movement results from a highly coordinated and complex mechanical interaction among bones, muscles, ligaments, and joints within the musculoskeletal system, which is regulated by the nervous system. Muscles produce tensile forces and generate moments at joints with relatively short lever arms, thereby ensuring both static and dynamic stability, while enabling precise limb control under gravitational and other external forces (Lu and Chang, 2012). At the core of motor control lies a sequence of transformations involving neural inputs, mechanical forces, and sensory feedback. Neural signals originating from the central nervous system (CNS) interact with mechanical forces generated by muscles, which subsequently drive movement and stabilize the musculoskeletal system (Nordin et al., 2017). External and internal forces, such as those exerted by the environment or between body segments, further modify movement patterns. Horak’s motion control theory emphasizes: “Normal motion control refers to the central nervous system by using existing and past information to transform neural energy into kinetic energy and enable it to perform effectively functional activities” (Horak, 1991). This process involves the interaction between the CNS and motor muscle tissue. For example, during a hand grip, the motor cortex sends commands through the motor conduction pathway, activating peripheral nerves and muscles in the upper body to initiate movement (Babiloni et al., 2008). Simultaneously, proprioceptive signals travel via the sensory conduction pathway to the spinal cord, brainstem, cerebellum, and partially to the cerebral hemisphere. Most of this sensory information reaches the brain’s sensory regions, where it is analyzed to adjust and regulate motor commands (Witham et al., 2011). Studying cortical-muscle function coupling offers insight into the communication between the cerebral cortex and muscle tissue, illustrating how the brain integrates movement and processes feedback from muscle contractions. In neurological disorders, this delicate interplay becomes disrupted, leading to altered motor patterns and functional impairments (Nordin et al., 2017). Understanding these disruptions requires a thorough analysis of both neurophysiological signals and biomechanical forces. In this sense, the assessment of neurological disorders demands a multidisciplinary approach, integrating neurophysiology, muscular control, and biomechanics of movement (Garro et al., 2021). This integrated approach is essential for advancing our understanding of motor dysfunctions and for developing targeted treatment strategies.

However, Bernstein (1967) found out that the main issue of motor control was redundancy. This theory proposes that the human body possesses redundant degrees of freedom, allowing multiple motor strategies to achieve the same goal. On the other hand, motor redundancy could be seen not as a “problem” but as a “bliss” of motor abundancy, as suggested by Latash (2012). This redundancy enables the motor system to be highly adaptable and capable of identifying efficient solutions under varying task demands. According to Latash, the equilibrium-point hypothesis combines the theory of motor control grounded in physical principles and consistent with neuromotor physiology (Feldman, 1986). This hypothesis was initially developed for single-muscle and single-joint systems, proposing that muscle activity can be controlled by setting a single parameter, the threshold of muscle activation relative to length. This approach applies to the principle of abundance, allowing the system to explore multiple patterns of neural activation to achieve the same task. In 2009, this theory was expanded into the referent configuration hypothesis for multi-joint actions (Feldman and Levin, 2009). This model, a hierarchical control system, defines a desired body action via subthreshold neural activity. Although this body’s action may not be fully achievable due to physical constraints, the body reaches an equilibrium state where muscle activations reflect the gap between actual and referent positions.

Among the theoretical approaches to motor control, optimal feedback control (Todorov and Jordan, 2002) has emerged as a prominent theory for explaining how the CNS manages the complexity of motor redundancy. Rather than viewing redundancy as a problem, this theory treats it as a feature that allows for flexible and efficient movement strategies. According to this model, the nervous system minimizes a cost function that balances task performance (i.e., minimizing error) with energetic efficiency (i.e., minimizing the variability or effort of control signals). Movements are continuously adjusted through optimized sensory feedback, enabling adaptability to changing conditions. Another theory on how the CNS manages motor redundancy is the uncontrolled manifold (Scholz and Schöner, 1999). Rather than attempting to control every individual joint or muscle, the CNS is thought to stabilize only those combinations of joint movements that are critical for achieving a specific motor goal, such as maintaining the center of mass or positioning the hand. Variability that does not interfere with the task outcome is allowed and even expected. This defines what is termed the “uncontrolled manifold.” Through quantitative analysis of the distribution of joint configuration variability across repeated task executions, it becomes possible to infer which task-specific variables are selectively stabilized by the CNS, thereby revealing their relative importance in the control hierarchy. For example, in a sit-to-stand task, Scholz and Schöner showed that the trajectory of the center of mass is tightly controlled, while the variability of joint motions that do not affect it remains relatively high. This suggests that the CNS organizes movement not by eliminating variability, but by shaping it in a task-relevant way.

The emerging concept of “neurobiomechanics” embodies an integrative approach, bringing together insights from functional anatomy, the physiology of the musculoskeletal and CNS, physics, and computer science (Ivancevic et al., 2015). By examining human movement under normal, optimal, and pathological conditions, neurobiomechanics aims to unravel the intricate mechanisms driving motor function and dysfunction, offering a comprehensive perspective on disorders like acquired brain injury and neurodegenerative diseases. What makes neurobiomechanics particularly valuable is its ability to investigate the complex interaction between neural signals and mechanical forces (Ivancevic et al., 2015). Neurological disorders are not only issues of neural dysfunction, but they also involve disruptions in the mechanical processes governing movement. The coordination between the brain, muscles, and skeletal structures is fundamental to motor control, enabling flexible and task-specific stabilization of movement. However, the aforementioned theories of motor control suggest that the nervous system exploits motor redundancy by stabilizing task-relevant variables, such as end-effector trajectories, equilibrium configurations, or referent positions, while allowing variability in dimensions that do not affect task performance. Disruptions at any level of this system can impair the ability to produce coordinated and goal-directed actions.

Traditional diagnostic approaches, focused on neurochemical, neuroimaging, and electrophysiological assessments, often overlook biomechanical factors. However, recent advances in this field highlight how mechanical forces can shape neural function, influencing disease pathology (Rocha et al., 2022; Pillai and Franze, 2024) and even guide rehabilitation outcomes.

Computational approaches are increasingly gaining traction within neurobiomechanics as powerful tools to explore the interaction between neural activity and musculoskeletal dynamics in both healthy and pathological conditions. Platforms such as OpenSim (NIH National Center for Simulation in Rehabilitation Research, Stanford, CA, USA)^1^ allow the estimation of joint forces and muscle activations based on kinematic and EMG data, offering a non-invasive way to study motor deficits and predict rehabilitation outcomes (Seth et al., 2011). MOtoNMS, a MATLAB (The MathWorks, USA)-based toolbox developed for MOtion data elaboration for NeuroMusculoSkeletal applications, facilitates standardized preprocessing of motion capture and EMG data, thereby streamlining their integration into neuromusculoskeletal simulations and enhancing the reliability of model-based analyses (Mantoan et al., 2015). On the neural side, simulators like NEURON^2^ (Hines and Carnevale, 1997) and Brian (Goodman and Brette, 2009) enable detailed modeling of neural circuits and the study of how changes in connectivity or signal transmission may affect motor control. More recently, platforms such as the NEUROmotor integration and Design (NEUROiD) (Iyengar et al., 2023) and the neuro-musculoskeletal flexible multibody simulation (NfMBS) (Geier et al., 2019) have contributed to the development of multiscale and modular environments that integrate neural, muscular, and skeletal models for simulating human movement under pathological conditions. These tools have been applied to study disorders such as Parkinson’s disease, stroke, and spinal cord injury, offering quantitative biomarkers and virtual environments to test treatment strategies. The integration of these computational tools into neurobiomechanical research supports a more systematic exploration of neural-mechanical interactions and helps bridge the gap between empirical observations and mechanistic understanding.

Recent studies, for instance, have shown that combining neurophysiological tools like electromyography (EMG) with biomechanical analysis can provide deeper insights into post-stroke motor recovery (Krauth et al., 2019; Kukkar et al., 2024). By coupling EMG with electroencephalography (EEG), researchers can objectively assess motor function during rehabilitation, offering more precise measurements of patient progress. In healthy individuals, the use of neurobiomechanical assessment was largely studied (Artoni et al., 2023; Hsu et al., 2023; Ortega-Auriol et al., 2023; Ke and Luo, 2024). This method was used to assess single or bimanual tasks, balance, and gait functions by exploring simultaneously neurophysiological and biomechanical data. In this sense, Ortega-Auriol et al. (2023) studied upper limb muscle synergies in healthy participants who executed an isometric upper limb task in synergy-tuned directions. In particular, the term “muscle synergy” refers to a set of muscles identified from experimental EMG data, characterized by consistent spatial and temporal patterns of activation, according to a certain model describing how these muscle groups are organized and contribute to the observed EMG signals (Berger and d’Avella, 2014; Cheung and Seki, 2021). Ortega-Auriol et al. measured cortical activity using EEG and muscle activity with EMG, revealing four distinct synergies from the multidirectional task, all showing significant intramuscular coherence (IMC) within the alpha band, particularly between muscles with high synergy weights. Additionally, a higher coherence strength correlation (CSC) was observed in the alpha band across high-weighted muscles within each synergy. However, no significant cortico-muscular coherence (CMC) was found between the motor cortex and synergy muscles, suggesting that, while shared neural input likely shapes these synergies, only specific ones may receive cortical modulation. Muscle synergies represent a way to reduce complexity by voluntarily reducing the dimensionality of the analyzed motor tasks (Tessari et al., 2025). It is worth noting that this is a positive connotation of their meaning. However, in the neurorehabilitation context, synergies can be referred to pathological movement patterns or undesired and involuntary movements on the paretic side, in the case of stroke (McMorland et al., 2015; Tessari et al., 2025).

Building on this concept, muscle synergy analysis, a method that decomposes multi-muscle surface EMG (sEMG) recordings into low-dimensional activation patterns, has gained popularity for its ability to provide meaningful insights into the neuromechanics of movement, both across different motor tasks and in the context of neurological impairments (Borzelli et al., 2024). In this sense, this concept supports the hypothesis that neurally driven muscle synergies are fundamental to human upper limb movement, with selective cortical influence on certain synergies. Another example can be the study of neuromuscular control of balance and posture, which is essential because both underpin fundamental human movement, impact quality of life, and are crucial for preventing injuries. Balance and postural control are especially critical in neurological disorders (e.g., acquired brain injury and neurodegenerative), where fall prevention can reduce severe injuries, such as fractures, reducing healthcare costs (Bryant et al., 2014; Cameron and Nilsagard, 2018; Bonanno et al., 2023). An innovative study by Ke and Luo (2024) has investigated neuromuscular coordination in healthy individuals, particularly the interaction between the cortex and muscles during varying levels of balance challenge. Using EEG and EMG in different balance paradigms, the study reveals that balance control involves shifts in brain-muscle communication, particularly in CMC patterns across the beta and gamma frequency bands. As balance demands increase, through conditions like closing the eyes or standing on unstable surfaces, the study found a stronger downward neural influence, indicating that the brain actively adjusts its control signals to the muscles. Concerning the rehabilitation field, knowing how the brain and muscles coordinate under different external conditions not only enhances our understanding of motor control but also underscores the importance of studying neuromuscular mechanisms to develop targeted rehabilitation interventions to recover motor functions. This aspect motivates a neurobiomechanical approach to study human behavior in both healthy and pathological conditions. Such an approach comprises the study of neural pathways involved in the activation of muscle synergies to achieve the goal of the movement and the biomechanics, which studies human movements through specific kinematic (trajectory and joint angles) and kinetic (muscular forces) elements (Ivancevic et al., 2015). However, this is only one of the many possibilities by which the CNS can control human motion (Feldman, 1986; Scholz and Schöner, 1999; Feldman and Levin, 2009; Latash, 2012, 2016). A recent work by Cheung and Seki (2021) provides valuable insight into the potential neural substrates of muscle synergies within both the central and peripheral nervous systems. They comprehensively reviewed the electrophysiological, anatomical, and behavioral evidence of muscle synergies, highlighting how they may be encoded at multiple levels of the motor hierarchy, including the spinal cord, brainstem, and motor cortex. Their analysis supports the view that synergies are not merely mathematical constructs but may reflect structured neural modules that contribute to coordinated motor output. Generally, traditional mathematical methods for extracting muscle synergies, such as non-negative matrix factorization or principal component analysis, can be used; however, they have intrinsic limitations due to the mathematical assumptions they impose, which may not fully capture the underlying neural mechanisms of motor control. Recent advances, such as the Synergy Expansion Hypothesis proposed by Tessari et al., suggest that synergies are not fixed modules but can be flexibly reorganized depending on the task demands. This framework offers a more biologically grounded and computationally robust approach to studying motor coordination, potentially addressing some of the methodological constraints of conventional synergy analysis.

Neurobiomechanics may offer valuable insights and objective methods for quantifying the relationship between neural and biomechanical aspects of human motion (Ivancevic et al., 2015). In the neurorehabilitation field, the neurobiomechanical assessment could be performed with multiple tools to assess both neurophysiology and biomechanics (e.g., EEG, EMG, wearable sensors), offering clinically relevant metrics for diagnosis, monitoring, and intervention planning (Taiar et al., 2022). However, there are several open questions regarding the implementation of this approach, related to the methodologies, the neurophysiological and biomechanical parameters extracted, and how this approach can be translated from research to clinical practice. In this review, we sought to explore the “neurobiomechanics” as a potential approach to investigate the neural and biomechanical aspects of human movement, trying to answer the following questions:

- Which technologies can perform a neurobiomechanical assessment in neurological patients?
- What are the key neurophysiological and biomechanical parameters?
- How can we translate this approach from research to clinical practice?

## 2 Search strategy

In this review, we selected evidence reporting neurophysiological and biomechanical assessment of movement in patients with neurological disorders (including stroke, Parkinson’s disease, multiple sclerosis, spinal cord injury, and cerebral palsy). We searched on PubMed and Scopus, without selecting a specific time range and searching for title/abstract, with combination of the following keywords: “neuromechanical assessment” OR “corticomuscular coherence” OR “corticomuscular coupling” OR “motor neurorehabilitation” OR “motor assessment” AND “neurological disorders” OR “parkinson’s disease OR “stroke” OR “cerebral palsy” OR “multiple sclerosis” OR “traumatic brain injury.”

To further investigate the role of computational modeling and simulation platforms in neurobiomechanical assessment, we performed a second targeted search using Google Scholar. The second search included the following additional keywords: AND “neuromusculoskeletal simulator” OR “biomechanical simulation” OR “musculoskeletal modeling” OR “computational neurorehabilitation.”

## 3 Overview of devices for neurophysiological and biomechanical instrumental assessment

In rehabilitation medicine, the evaluation phase is the first step to build a personalized rehabilitation pathway, according to patients’ needs, strengths, and residual functions. This is why it is important to have a global, but also quantitative and objective idea of the movement patterns of the patient, which is strictly individual due to diagnosis, etiologies, timing and location of the injury. In this context, quantifying subject-to-subject variability is a critical component of both clinical and laboratory investigations. Moreover, variability is often analyzed to assess motor control across different scenarios, providing insights into how the CNS simplifies muscle coordination by reducing the dimensionality of control (Rosati et al., 2017; Pale et al., 2020; Zhao et al., 2021).

The clinical assessment of patients’ physical and physiological health is fundamental for both physicians and physiotherapists to determine if the exercise has an influence and, ultimately, to modify their follow-up rehabilitation program, according to their specific needs. To this aim, a range of technologies, spanning from conventional methods (e.g., EEG, EMG) to innovative tools (e.g., robotic devices, markerless motion capture), can complement the clinical evaluation, including:

### 3.1 Neurophysiological investigation systems

EMG records the electrical activity of muscles and is used to assess muscle activation patterns, strength, and coordination. SEMG is widely utilized in neurorehabilitation due to its non-invasiveness, ease of application, and ability to provide real-time insights into muscle activity, aiding in both diagnosis and treatment monitoring (Disselhorst-Klug and Williams, 2020). However, sEMG can present several factors that can produce different types of noise signals. For example, proper skin preparation, including exfoliating, shaving, cleansing, and applying conductive gel, is essential for reducing electrode impedance and noise at the electrode-skin interface, thereby improving the quality of sEMG recordings (McManus et al., 2020). In addition, crosstalk, an undesired signal interference caused by overlapping electrical activity from neighboring muscles, can make it difficult to accurately identify the signal origin, especially when adjacent muscles are simultaneously active (Chowdhury et al., 2013; Pilkar et al., 2020). Although it can be minimized through careful selection of electrode placement, size, and spacing, crosstalk remains influenced by physiological factors such as subcutaneous fat and electrode orientation (Chowdhury et al., 2013). Moreover, proper electrode placement, following the SENIAM guidelines (Hermens et al., 2000), is essential, with electrodes typically aligned along the direction of muscle fibers and positioned on the muscle midline. Importantly, no software can compensate for fundamental operator errors, such as incorrect electrode placement or inappropriate filtering (Campanini et al., 2020; McManus et al., 2020).

Nevertheless, sEMG can be used to obtain information regarding muscle activation during a movement, giving an idea of functional alteration. In neurorehabilitation, the application of sEMG can be suitable for the detection of co-activation of agonist and antagonist muscles, providing information about neuromuscular coordination control among muscles (Disselhorst-Klug and Williams, 2020; Al-Ayyad et al., 2023). In this sense, the concept of muscle synergies can simplify motor control without significantly affecting performance, as demonstrated by several authors (d’Avella et al., 2003; Ting and McKay, 2007; Bizzi et al., 2008; Berger and d’Avella, 2014). The clinical relevance of muscular coordination is particularly evident in individuals with neurological conditions, where analyzing muscle activation patterns during reaching/grasping movements for the upper limb, or during gait for the lower limbs. A recent work (Avni et al., 2024) has demonstrated that both flexor synergy intrusion and muscle weakness contribute substantially to abnormal reaching kinematics in the sub-acute post-stroke phase. Specifically, 3D kinematic analyses revealed that synergy intrusion particularly affects out-of-synergy movements, and that both weakness and synergy explain a significant proportion of variance in clinical and functional scores. Taken together, these findings highlight the importance of moving beyond traditional clinical scales toward a more detailed characterization of neurological deficits through quantitative kinematic analysis of movements (Kwakkel et al., 2017). After a stroke, grasping movements are also impaired, with delays in palmar arch modulation and finger pre-shaping, as well as slower, less precise grasp aperture opening (Roby-Brami et al., 2021). Despite these deficits, stroke survivors rely on compensatory mechanisms within the sensorimotor system. These allow alternative finger coordination strategies, such as delayed metacarpophalangeal flexion, to adapt grasping to different object shapes, even when typical finger movements are limited (Raghavan et al., 2006, 2010). Interestingly, in another study (Lin et al., 2023), the authors demonstrated that proximal and distal upper limb motor systems can be selectively and independently affected by acute stroke, leading to distinct clinical syndromes and functional outcomes. In this study with 141 patients after stroke, the authors found that proximal and distal motor deficits are dissociable, with distal motor control often relatively preserved in many patients. Notably, patients with preserved distal function showed better recovery outcomes both in the acute phase and after 90 days. Neuroanatomically, proximal deficits were linked to widespread damage in descending motor pathways, while distal deficits were specifically associated with injury to the primary motor cortex. These findings highlight the distinct anatomical and functional organization of proximal vs. distal motor systems and their differential vulnerability and impact following stroke.

Although EEG provides high temporal resolution, it has limitations. For example, in some systems, a limited number of channels can lead to source mis-localization, affecting the accuracy of Source Electrical Imaging (ESI) (Formica et al., 2025). Moreover, the EEG system is susceptible to biological factors, such as muscular components, movements and extra biological artifacts, such as eddy current (Gorjan et al., 2022). For these reasons, EEG acquisitions need qualified staff in the best choice of measure cap, montage, and in the management of artifacts during acquisitions (Fiedler et al., 2022). EEG devices are innovative because they can be triggered by external systems, such as E-Prime (Psychology Software Tools, Inc.; PA, USA), to analyze brain oscillations in response to specific events (MacWhinney et al., 2001). However, interfacing EEG with other neurophysiological tools can be challenging, especially when these systems use wireless methods for data acquisition and transmission, which may introduce excessive noise into the EEG recordings (Gorjan et al., 2022).

Moreover, EEG systems transmit signals via wired connections attached directly to their amplifiers, which limits the ability to perform large upper limb movements or to record cerebral electrical activity during walking (Mundanad Narayanan et al., 2021). Recent technological advances are addressing interface limitations by developing wireless EEG systems (Mundanad Narayanan et al., 2021). EEG acquisition can be conducted either by directly analyzing the electrical potential differences recorded at each electrode or by adding an intermediate step involving the reconstruction of cortical sources to obtain the temporal and spectral patterns of the brain’s electrical field generators (Astolfi et al., 2007; Scano et al., 2023). Analyzing the connectivity of the brain aims to identify the areas that are synchronously active both at rest and during a given task. For example, while beta activity increases with motor practice and returns to baseline after rest, its amplitude does not depend on specific movement features. In contrast, gamma activity varies in proportion to movement speed and distance. In this context, Tatti et al. (2023) found that the overall amplitude of gamma activity decreased following motor practice as well as after periods of sleep or quiet rest. These findings suggest an effect partially associated with participants’ subjective fatigue.

Additionally, connectivity in brain regions linked to attention also decreased after both practice and rest. These results confirm that gamma activity is involved in movement control but suggest that the reduced amplitude is more likely due to mental fatigue or more automatic movement, rather than changes in brain plasticity. To analyze brain connectivity, two types of approaches can be distinguished: functional and effective analysis (Cao et al., 2022). In functional analysis, the functional network organization is examined; in effective analysis, the directionality and causal influence between structures are also assessed. Some authors combined EEG/EMG analyses, demonstrating that this investigation can be used to evaluate the integrity of the neuromuscular system in subjects with spinal cord injury (Leerskov et al., 2020) or to study the sensorimotor cortex in patients with cerebral palsy (Short et al., 2020). Beyond their role in active control of movement and brain oscillations are increasingly recognized for their involvement in learning processes. The acquisition or improvement of sensorimotor skills relies on mechanisms of neural plasticity that support the formation and refinement of motor synergies (Tatti and Cacciola, 2023). In this context, other authors have quantified the coherence function between the EEG and EMG signals, which is named as CMC (Liu et al., 2019). CMC is considered a useful method to study the mechanism of the cerebral cortex’s control of muscle activity. It demonstrates the functional link between the cortex and muscles during sustained contractions. CMC originates from communication within the corticospinal pathways, connecting the primary motor cortex to the muscles (Liu et al., 2019).

Furthermore, Mobile Brain/Body Imaging (MoBI) consists of a simultaneous registration of brain activity, using EEG, and muscle activity, with EMG signals. This approach can be used to record and analyze brain dynamics and human movement under naturalistic conditions. Regarding brain imaging, only EEG and functional near-infrared spectroscopy (fNIRS) have sensors suitable for measuring brain activity during active movement (Jungnickel et al., 2019). This combined approach could be a new solution to investigate the potential communication between supraspinal and subcortical sites with concurrent kinematic events (De Sanctis et al., 2020). This approach introduces novel, important opportunities enabling the investigation of the role of the CNS during movement. A MoBI set-up consists of a flexible multimethod approach to record human movement in unrestricted conditions, by using lightweight body sensors concurrently with brain activity (e.g., by means of EEG). The essence of the MoBI approach would require not only a mobile brain recording device but also simultaneous (and thus precisely triggered in time) other measurements (e.g., visual, auditory, and tactile stimulation as inputs, motion capture, force measures, or scene and gaze tracking as outputs) (He et al., 2018; Delaux et al., 2021) to explore key aspects of the human locomotion linked to brain behavior (Delaux et al., 2021). Examples of such mobile wearable body sensors could be wireless sEMG, 3D motion capture, inertial measurement units (IMUs), foot plantar pressure measurement systems, and/or eye tracker devices.

### 3.2 Biomechanical investigation systems

The study of movement biomechanics involves the analysis of kinematic and kinetic components, which can be evaluated through specific devices capturing the multiple aspects of movement biomechanics. Optoelectronic systems consist of marker-based motion capture systems (MoCap). These systems are considered as “gold standard” for motion detection (Romero-Flores et al., 2024; Scataglini et al., 2024), since they use infrared light to accurately estimate the 3D position of a set of active or passive markers, by triangulation from multiple cameras (Ceseracciu et al., 2014). Passive markers are covered by photo-reflective materials that reflect infrared light, while active markers emit infrared light. In particular, the markers are positioned on anatomical landmarks in correspondence with the joints involved in the analysis to track all the human motion features with high accuracy (Zago et al., 2020). MoCap allows 3D body model reconstruction; however, it is time-consuming for the complexity of the setup, and patients are not free to move easily due to the presence of multiple markers. These systems are largely used to perform 3D motion analysis, since they provide, through *post hoc* analysis, detailed information about joint mobility, coordination, and abnormal movement patterns. Despite the large use of MoCap systems for research purposes, these are still not implemented in clinical practice, due to their high costs and set-up complexity (Pardell et al., 2024). Some of the most adopted MoCap systems are the Vicon system (Vicon Motion Systems, Oxford, United Kingdom) and the Optitrack system (Corvallis, OR, USA), consisting of multiple infrared cameras for kinematic and spatiotemporal movement analysis. Optoelectronic systems can be used for upper limb (De Pasquale et al., 2024) as well as for lower limb (Bonanno et al., 2024) motion analysis to detect quantitatively movement alterations, including compensations and pathological synergies, and to monitor progress after the rehabilitation intervention.

Markerless motion capture (MMC) systems are growing rapidly in the field of neurorehabilitation. These systems avoid the need for marker placement during motion analysis. Different from MoCaps, MMC allows for reducing time-consuming marker placement and costs related to the equipment. Generally, MMC requires a standard camera, such as an RGB or infrared sensor, to register human movements. For more accurate 3D motion tracking, multiple cameras can be used from different angles (Wade et al., 2022). Once the video is captured, pose estimation algorithms process the footage using artificial intelligence systems like OpenPose (CMU-Perceptual-Computing-Lab/openpose, 2025), MediaPipe (google-ai-edge/mediapipe, 2025), or DeepLabCut (DeepLabCut/DeepLabCut, 2025). These systems analyze key body points, such as joints and limbs, by applying deep learning techniques, such as Convolutional Neural Networks (CNNs), to accurately detect skeletal structures. In this way, these systems allow the capture of a more lifelike human motion in the environment, in a more natural way. Through these features, they can be used with more portable and low-cost sensors compared to marker-based multi-camera systems (Lam et al., 2023). Recent approaches (Cotton et al., 2023; West et al., 2023) have improved accuracy by using dense key point sets trained on multiple datasets, such as MeTRAbs, which better capture critical areas like the pelvis and torso. Additionally, new methods use neural networks to reconstruct smooth and anatomically consistent 3D trajectories from video, leading to more reliable inverse kinematic analysis. These advances make MMC suitable for real clinical use, allowing quick, detailed movement analysis in settings like rehabilitation hospitals, even with patients who use assistive devices or have complex motor impairments.

Wearable sensors (WS) are often based on IMUs, which comprise accelerometers, gyroscopes, and magnetometers. These devices are portable and low-cost, and they can easily be attached to different body parts (e.g., ankles, wrists, or trunk) (Iosa et al., 2016). WS can be used to assess movements during activity of daily living, enabling unsupervised assessment (Wang and Choi, 2020). WS can be used to monitor patients at home, after discharge from the hospital, to guarantee continuity of care (van Melzen et al., 2023). In clinical settings, WS can be used to measure kinematics of upper and/or lower limbs outside laboratory settings, promoting real-world evaluations (Lang et al., 2020). However, compared to optoelectronic systems, IMUs are considered less accurate, since metal objects in the evaluation environment cause electromagnetic interference and significant distortions (van Schaik and Dominici, 2020; Zhou et al., 2020). On the other hand, the advantage in using IMUs systems is the absence of occlusions (because there are no barriers between sensors and transmitter), the large capture volume, and the availability of position and orientation data without post-processing analysis (Wirth et al., 2019). These systems offer portability, being lightweight and wireless, adaptable to diverse environments, albeit within proximity to a receiver and acquisition system (De Marchis et al., 2023). They combine cost-effectiveness and minimal recording latency, facilitating real-time applications (Ali et al., 2025). This is why they are often used for recording in real-time situations. However, inherent limitations, notably drift accumulation leading to estimation inaccuracies over time, impel reliance on external references for absolute accuracy (Takeda et al., 2014). To address this, a method was proposed that resets velocity estimates during the foot’s stance phase to constrain error accumulation (İkizoğlu et al., 2025). Using IMUs placed on the foot and knee, the approach achieved high accuracy in short-path gait analysis (error ~ 0.8%), without needing complex calibration setups. While effective for the foot, additional correction strategies were required for the knee, highlighting the importance of adaptive, segment-specific compensation techniques in inertial tracking systems. According to a recent systematic review by Gu et al. (2023), the use of IMU-based MoCap in rehabilitation assessment is quite mature. Several articles (Patel et al., 2012; Lefeber et al., 2019; Weygers et al., 2020) have validated the acceptable accuracy of the IMU sensor compared to the gold standard optical MoCap in a rehabilitation context.

In MoCap research, eye tracking technology provides objective insights into decision-making, attention, and cognitive load in medical settings (Pauszek, 2023). Most eye trackers, regardless of make or model, use the pupil-corneal reflection technique to determine an observer’s point of gaze. This method relies on cameras, illuminators, and image-processing algorithms. Cameras capture high-resolution images of the eye multiple times per second, while near-infrared illuminators project a light pattern onto the eyes. This light, invisible to the human eye but detectable by the cameras, creates reflection patterns on the pupil and cornea. These reflections serve as reference points for algorithms to calculate gaze angles and map them onto the external world. The gaze data is then integrated with a video feed from an outward-facing camera or monitor (Pauszek, 2023).

In the field of kinetics, force sensors are used to study the interaction forces between the body and its environment. For instance, some authors have used force sensors to record the maximum voluntary isometric contraction (MVC) of the hand and force maintenance during contractions under constant load (Lai et al., 2016). Other authors have estimated the upper limb’s MVC from sEMG signals, as muscle activation has also been used to estimate the end-point force generated by a human operator (Borzelli et al., 2023).

Other authors used this kind of sensor to obtain pressure foot evaluation during gait (De Sanctis et al., 2020; Kukkar et al., 2024). In addition, force sensors were used to detect ground reaction forces during gait analysis, as well as in dynamic posturography, to measure center of pressure (COP) (Kukkar et al., 2024).

Lastly, robotic devices that deliver motor rehabilitation sessions for upper and lower limbs can also serve as instrumental evaluation devices, as they are equipped with sensors (e.g., digital goniometers and force sensors) that provide kinematic and kinetic analyses. Robot-aided motion analysis (R-AMA) can be used to quantitatively register spatial-temporal parameters of movement and allow personalization of the rehabilitation path according to patients’ needs (Bonanno and Calabrò, 2023). This is particularly true for devices whose assessment protocols have been validated in terms of reliability. For example, Bolliger et al. (2008) demonstrated the inter- and intra-rater reliability of Lokomat (Hocoma AG, Volketswil, Switzerland) for measuring maximal voluntary isometric muscle force. The authors suggested that this method is a valuable tool for documenting and monitoring the rehabilitation process in patients using a robotic device, as it enables a personalized approach to create a tailored patient profile that includes a precise physiotherapy program.

## 4 Neurobiomechanical assessment approach in patients affected by acquired brain injury

Acquired brain injury (ABI) is an umbrella term that indicates patients affected by an acute CNS lesion, commonly derived from cerebrovascular and/or traumatic causes. This condition can affect people of all ages, from children to adults and older individuals, causing sensory loss, muscle weakness, spasticity, and cognitive alterations. Altogether, these factors impact movements, including reaching tasks and manual strength as well as gait and postural stability (Goldman et al., 2022). This aspect motivates a neurobiomechanical approach to studying human behavior in both healthy and pathological conditions (Table 1). This approach comprises the study of neural pathways involved in the activation of muscles to achieve movement goals and the biomechanics, which studies human movements through specific kinematic (trajectory and joint angles) and kinetic (muscular forces) elements (Ivancevic et al., 2015).

**Table 1:** Summary of the selected studies on stroke and cerebral palsy population.

First author and year of publication	Function/body target	Study population and sample characteristics	Assessment methodologies	Parameters analyzed	
				Neurophysiological	Biomechanics
Belardinelli et al. (2017)	Upper limb/hand flexion-extension movements.	8 right chronic post stroke patients, consisting of 7 males and 1 female (mean age 57 ± 11 years).	MEG (instead of EEG) EMG detected muscle activity in the forearm, specifically in the Extensor Digitorum Communis (EDC), which was used for the final analysis. MRI was used to acquire structural brain images with a voxel resolution of 1 × 1 × 1 mm and construct three-dimensional models for cortical source analysis. Robotic hand orthosis (Amadeo)	MEG: CMC in the same frequency band to assess the synchronization level between brain activity and muscle activity. MRI images: the spatial distribution of coherence sources was analyzed, identifying the main cortical areas involved. EMG: activation intensity before and after the intervention of EDC, ECU, FCR, and RMS.	Robotic hand orthosis movements (flexion/extension).
Delcamp et al. (2023)	Upper limb (elbow flexion-extension movements).	24 chronic post-stroke subjects (mean age 57 years), comprising 20 M and 4 F. Lesions were distributed between the right and left hemispheres.	EMG from the flexor muscles (BB and BR) and the main extensor muscle (TB) during active elbow extension movements. EEG was performed using a 64-electrode system. Motion capture systems (OptiTrack) were used to record the kinematics of elbow extension movements, with a sampling frequency of 125 Hz.	CMC and IMC analyze the correlation between EEG (cortical) and EMG (muscular) signals in the time-frequency domain, capturing the oscillatory dynamics between the brain and muscles.	Elbow angle during active extension and the movement velocity.
Fang et al. (2009)	Upper limb/reaching movements.	21 stroke patients (16 M and 5 F, with a mean age, 59.57 ± 8.61) who had persistent dyscoordination of the upper limb. 8 healthy controls (5 M and 3 F, with a mean age 60.62 ± 6.25)	EEG with a 64-channel NeuroScan system EMG from AD, BB, TB muscles. To standardize movement and record kinematic data, a robotic device (InMotion2) which collected detailed data on trajectory and applied force.	EEG–EMG coherence	The lateral deviation, which reflects the patients’ difficulty in maintaining the desired trajectory during the movement. Movement Duration Applied Force
Fauvet et al. (2021)	Upper limb/elbow extension movements.	8 healthy control volunteers (43 ± 21 years, three F) and 17 chronic phase post-stroke patients (58.2 ± 12.7 years, four F and 13 M).	EEG for continuous recording with a 64-channel system (ActiveTwo System, Biosemi). Surface EMG recorded from triceps brachii, biceps brachii, brachialis, and brachioradialis. Upper limb kinematic sensors recording at 125 Hz using an eight-camera infrared system (model S250e, Optitrack). Reflective markers placed on anatomical landmarks (e.g., lateral epicondyle, ulnar styloid, second metacarpal). EEG, EMG, and kinematic data were synchronized via TTL pulses generated by the Biopac MP150 system.	CMC focusing on the beta frequency band (13–30 Hz). Average CMC: Calculated over the entire movement, divided into acceleration and deceleration phases.	Elbow extension angle. Peak angular velocity. Smoothness of movement.
Guo et al. (2020)	Upper limb/distal finger movements.	14 chronic stroke subjects, with a mean age of 56.5 ± 9.5.	EEG: A 64-channel. EMG: Surface EMG was recorded from four muscles: extensor digitorum (ED) and flexor digitorum (FD) (distal muscles). Triceps brachii (TRI) and biceps brachii (BIC) (proximal muscles). Robotic hand orthosis. EEG and EMG signals were synchronized using a DAQ card (NI, USB-6009, 14-bit multifunction DAQ USB) at a sampling frequency of 1,200 Hz.	CMC was measured in the Beta band (13–30 Hz). The peak CMCoh was calculated for the extensor and flexor muscles of the fingers (ED and FD) as well as for the proximal muscles (TRI and BIC) during finger extension and flexion tasks. EMG: Co-contraction Index (CI).	Robotic hand orthosis flexion/extension movements
Krauth et al. (2019)	Upper limb/wrist movements.	7 healthy volunteers, 5 acute and subacute post-stroke patients.	EEG: A 64-channel EMG using bipolar electrodes placed on the wrist extensor muscles (2 electrodes in each hand). Resting conditions: Recordings were also made during periods of rest for comparison.	CMC	Isometric strength and movement attempts Wrist movement assessed using the FMA scale.
Lai et al. (2016)	Upper limb/thumb flexion.	15 healthy adults (8 females, 24.0 ± 1.5 years old) 15 stroke survivors.	EMG from the tenar eminence muscles to assess muscle activity. EEG from the primary motor area contralateral to the stimulated side. Force meters, a force-sensing device used to record the maximum voluntary isometric contraction (MVC) of the thumb and force maintenance during contractions under constant load.	EEG–EMG coherence.	Force deviation from target of 50% of maximum voluntary contraction (MVC).
Zhou et al. (2021)	Upper limb/distal finger movements.	14 subjects with stroke (mean age 56.5 ± 9.5 years) (3 M and 8 F) and 11 healthy subjects (mean age 50.6 ± 16.8 years) (3 M and 8 F).	EEG: 64-channel EEG electrode system to record brain activity in the sensorimotor cortex. Four-channel EMG electrodes (Blue Sensor N, Ambu Inc.) were applied to the upper limb muscles estensor digitorum (ED) flexor digitorum (FD), triceps brachii (TRI), biceps brachii (BIC). A robotic hand orthosis was used to fix the wrist and finger posture, standardizing the position of the upper limb during the motor task.	CMC measured in both descending (EEG → EMG) and ascending (EMG → EEG) pathways to assess the interaction between the sensorimotor cortex and muscles during fine motor control. Relative muscle strength.	Wrist and finger posture, position of the upper limb during the motor task.
Bao et al. (2021)	Lower limb/pedaling.	Ten chronic stroke subjects, 5 females, 5 males; age: 57.1 ± 11.6 years; time since stroke: 4.3 ± 2.3 years.	EEG during isometric contraction tasks, 128-channel. EMG placed on RF, VL, VM. NMES-pedaling training system set to 2 kHz.	EMG analysis: normalized muscle activation ratio, force steadiness, adjust NMES intensity, ensuring a personalized training regimen that optimally stimulates the muscles. EEG analysis: CMC was analyzed in the Beta (13–30 Hz) and low Gamma (30–45 Hz) bands, linked to motor control and isometric contractions.	Kinetic parameters included torque balance during pedaling, and pedaling speed (10–25 RPM) adjusted based on muscle activation ratio for personalized training.
Xu et al. (2023)	Lower limb/ankle dorsiflexion.	Twelve stroke patients (4 females, mean age ± 55.58 ± 11.81 years old) were recruited. The study also recruited 15 healthy controls with similar ages (8 females, mean age ± standard deviation: 49.20 ± 10.26 years old).	EEG. EMG from Tibialis Anterior (TA), Lateral Gastrocnemius (LG) and Medial Gastrocnemius (MG) muscles.	CMC.	Fugl-meyer assessment lower limb
Kukkar et al. (2024)	Lower limb/balance.	14 post-stroke patients (age: 61.93 ± 8.97 years, mean ± SD; range: 37–70) and 10 healthy adults (age: 55.3 ± 8.65 years, mean ± SD; range: 35–65).	EEG-64 channels (Brain Products GmbH, Germany; 1,000 Hz). EMG (Delsys Trigno EMG System, Boston, MA) from tibialis anterior (TA), gastrocnemius medialis (GM) and soleus (SOL) as distal leg muscle group, and rectus femoris (RF) and biceps femoris (BF). Computerized dynamic posturography (CDP) was assessed using a commercially available CDP force platform (Neurocom Balance Master, Natus Medical Incorporated, Pleasanton, CA).	CMC across different frequency bands (delta, theta, alpha, beta, gamma).	Postural Stability Metrics, including RMS COP, COP path length (PL), and COP velocity (RMSCOPv), to quantify postural performance.
Short et al. (2020)	Lower limb/Treadmill walking.	9 children with unilateral CP (7 F, 2 M; with a mean age of 16.0 ± 2.7 years) and 12 with TD (8 F, 4 M; age: 14.8 ± 3.0 years).	64-channel wireless EEG system captured brain activity during walking and standing. EMG from TA, MG, soleus (SOL), long peroneus (PL), rectus femoris (RF), vastus lateralis (VL), biceps femoris (MH) and long flexor hallucis (HL). Vicon motion capture system with 10 cameras and reflective markers on anatomical points of the pelvis and lower limbs. Kinematic data recording at 100 Hz to segment the gait cycle and synchronize with EEG and EMG.	EEG spectral power modulation.	Support time; Walking speed and stride length; Cadence.
Forman et al. (2022)	Lower limb/ankle dorsiflexion.	14 M and 7 F with a mean age of 37.6 years (± 10.1). Neurologically intact (NI) group 10 participants were recruited, 4 male, 6 female.	EEG recording using a 64-channel helmet (BioSemi, The Netherlands). EMG recording of muscle activity using surface electrodes applied to the tibialis anterior (agonist) and soleus (antagonist) muscles.	Correlation between EEG and EMG activity (forward and reverse).	Maximal torques and MVC fatigue test.

The table reports the characteristics of the included sample, target function, technological equipment for the neurobiomechanical assessment, and neurophysiological and biomechanical parameters.

M (Male), F (Female), NMES (Neuromuscular electrical stimulation), CMC cortico-muscular coherence; Magnetencephalography (MEG), RMS (Root Mean Square), Extensor Digitorum Communis (EDC), Extensor Carpi Ulnaris (ECU), Flexor Carpi Radialis (FCR); Intermuscular coherence (IMC); anterior deltoid (AD), biceps brachii (BB), and triceps brachii (TB); Brachioradialis (BR); electrical stimulation (ES); Fugl-Meyer (FMA); cerebral palsy (CP); healthy controls (HC); Typical development (TD); Brain magnetic resonance imaging (MRI); Transcranial Magnetic Stimulation (TMS); central motor conduction time (CMCT); Functional Corticomuscular Coupling (FCMC); Direct cortico-muscular coherence (dCMC); transcranial magnetic stimulation (TMS); somatosensory evoked potentials (SEPs); Electro-oculography (EOG).

### 4.1 Upper limb function in ABI

Guo et al. (2020) employed CMC and EMG analyses to investigate the corticomuscular coordination patterns associated with compensatory proximal upper limb activity during distal movements in individuals with chronic stroke. They recorded EEG data from the sensorimotor region and EMG signals from the extensor digitorum (ED), flexor digitorum (FD), triceps brachii (TRI), and biceps brachii (BIC) to assess CMC peak values in the beta band. EMG parameters, including the activation level and co-contraction index (CI), were analyzed to assess compensatory muscular patterns in the upper limb. These authors found that stroke patients showed significant shifts in CMC from the ipsilesional to the contralesional side in proximal upper limb muscles, while distal muscles showed central region coherence. In addition, stroke patients demonstrated higher EMG activation levels and CIs in the TRI and BIC compared to controls, indicating increased compensatory activity in proximal muscles during both extension and flexion tasks. Significant differences in CMC were observed in ED and FD muscles during finger extensions between stroke patients and controls, highlighting distinct compensatory patterns in stroke recovery. However, the study by Zhou et al. (2021) further investigates the neural pathways and specific timing of these interactions during fine motor control of finger extension by analyzing directed CMC (dCMC). While the study by Guo et al. (2020) confirmed the cortical origin of proximal compensation, Zhou et al. (2021) extended these findings by examining the directionality of neural pathways, differentiating descending signals (brain to muscle) from ascending feedback (muscle to brain). It reveals a pronounced descending dCMC in the proximal upper limb (BIC and TRI) among stroke patients, which differs from controls, who display this coherence dominantly in the distal upper limb (ED and FD). Interestingly, the study of Zhou et al. (2021) suggests that EMG stability can indicate the quality of motor control. In stroke patients, the affected limbs show lower EMG stability and stronger ascending feedback from the distal muscles, likely as compensation for impaired fine motor control. In addition, Zhou et al., by examining the timing of descending dCMC, identified a significant delay in corticomuscular conduction time in the ED muscle of the affected limb compared to both the unaffected and control limbs.

Krauth et al. (2019) further support the potential of CMC as a measurable biomarker for motor recovery by showing that coherence rises as patients regain motor function, distinguishing the brain’s adaptation patterns over time. In particular, they observed that patients demonstrated larger, more bilaterally distributed cortical involvement compared to healthy controls. This expanded cortical distribution may signal compensatory neural reorganization to support motor recovery.

Both Krauth’s and Zhou’s studies imply that certain cortical regions play critical roles in functional compensation and recovery after stroke. The finding that EEG–EMG coherence patterns differ spatially and temporally between patients and controls suggests that CMC could guide targeted, real-time rehabilitation strategies aimed at specific cortical areas to enhance motor recovery (Krauth et al., 2019). However, Makin and Krakauer (2023) offer a different perspective, as they propose that these neural pathways already exist before injury but remain largely inactive. Rehabilitation may not create new pathways, but rather facilitate the activation of these pre-existing, residual connections. In this view, the temporary expansion of cortical maps observed after intensive rehabilitation does not necessarily indicate functional reorganization but may instead be an epiphenomenon of the underlying process driving motor recovery.

Another study (Fauvet et al., 2021) further investigated CMC’s role in regulating both agonist and antagonist muscle activity during voluntary movements, specifically focusing on its modulation in real time. The authors found that CMC dynamically adapts in real time between the cortex and muscles, particularly by continuously integrating both afferent (sensory) and efferent (motor) information. This finding supports the idea that CMC reflects the brain’s adaptive control in motor tasks. While previous research highlighted higher proximal CMC in post-stroke compensations (Krauth et al., 2019; Guo et al., 2020; Zhou et al., 2021), this study found that stroke patients exhibit higher instantaneous CMC in antagonist muscles during the acceleration phase of elbow extensions, a pattern not seen in healthy controls. The increased antagonist CMC observed by Fauvet et al. may reflect cortical-level adaptations, which could coexist with or contribute to the altered synergy patterns, such as merging, reported in synergy-based analyses of post-stroke motor control (Cheung et al., 2012).

Regarding the biomechanics of the upper limb movements, Fauvet et al. found that the peak angular velocity of the upper limb was significantly reduced, and the smoothness was altered, compared to healthy controls. Taken together, both neurophysiological and biomechanical information can give a clear idea of the specific movement deficits of this patient population, allowing for personalized rehabilitation therapy. Moreover, Fang et al. (2009) provided other insights into how reduced brain-muscle connectivity may contribute to upper-limb motor deficits in post-stroke patients. These authors found that in post-stroke patients, EEG–EMG coherence in the gamma band (30–40 Hz), which is associated with cognitive functions (e.g., motor planning and information integration), was almost absent, suggesting reduced communication between the brain and muscles. The coherence in the beta band (20–30 Hz), related to motor control and submaximal force production, was reduced in patients compared to controls, although the effect was less pronounced than in the gamma band. In addition, they found a reduced coherence in the agonist muscles (AD and TB) in post-stroke patients, particularly in the gamma band, with a lesser involvement of the BB in patients compared to controls, indicating possible difficulties in movement coordination. According to the authors’ results, post-stroke patients manifested a higher lateral deviation trajectory, indicating difficulty in maintaining the desired trajectory, and they required more time to complete the reaching task.

Fang et al. provided other insights into how reduced brain-muscle connectivity may contribute to upper-limb motor deficits in post-stroke patients. These authors found that in post-stroke patients, EEG–EMG coherence in the gamma band (30–40 Hz), which is associated with cognitive functions (e.g., motor planning and information integration), was almost absent, suggesting reduced communication between the brain and muscles. The coherence in the beta band (20–30 Hz), related to motor control and submaximal force production, was reduced in patients compared to controls, although the effect was less pronounced than in the gamma band. In addition, they found a reduced coherence in the agonist muscles (AD and TB) in post-stroke patients, particularly in the gamma band, with a lesser involvement of the BB in patients compared to controls, indicating possible difficulties in movement coordination. According to the authors’ results, post-stroke patients manifested a higher lateral deviation trajectory, indicating difficulty in maintaining the desired trajectory, and they required more time to complete the reaching task. Delcamp et al. (2023) found that not only is CMC altered in post-stroke patients, but also intermuscular coherence (IMC), which represents the shared central drive to multiple muscles. In particular, Delcamp et al. recorded EEG and EMG data from both the elbow flexor and extensor muscles in both healthy and post-stroke individuals. CMC and IMC values were calculated in the time-frequency domain for each limb in both the stroke and control groups. A significant correlation was observed between CMC and IMC in both the paretic and non-paretic limbs of post-stroke participants. This correlation suggests that, in stroke patients, there is a form of motor control simplification, likely beyond previously proposed cortical and spinal explanations. When central-peripheral communication increases in these individuals, it becomes less modulated and more uniformly distributed across the muscles involved in movement. This motor control simplification may offer new insights into the plasticity and reorganization of the neuromuscular system following stroke. Other authors recorded MEG and EMG signals while patients tried to open and close their paralyzed hand, both before and after a four-week robotic training. The goal was to test whether the training could increase CMC in patients with severe and lasting impairments.

Belardinelli et al. (2017) used simultaneous MEG/EMG recordings along with magnetic resonance imaging (MRI)-based individual models to analyze CMC. This multimodal approach allowed researchers to map cortico-muscular connectivity to finger extensors in stroke patients, both before and after a rehabilitation training program. After the training, patients’ upper extremity Fugl-Meyer Assessment (FMA) scores showed a significant improvement, increasing from 16.23 ± 6.79 to 19.52 ± 7.91 (*p* = 0.0015). All patients exhibited significant increases in CMC within the beta frequency range, showing a distributed pattern across both hemispheres, although with high variability between individuals. The observed CMC changes did not correlate with motor impairment severity, the degree of motor improvement, or lesion volume. This is in line with previous findings by Rossiter et al. (2013), who also reported a lack of correlation between impairment levels and CMC patterns, including in patients with varying degrees of motor deficits and at different post-stroke stages. Nevertheless, the findings by Belardinelli et al. support the hypothesis that the contralesional hemisphere can serve as a source of coherent descending cortical input to muscles involved in functional motor tasks following stroke. Importantly, the observed CMC increases cannot be attributed solely to changes in EMG amplitude, as EMG root mean square (RMS) values varied across patients without a consistent pattern. Collectively, these results suggest that CMC, particularly in the beta-band within premotor and contralesional areas, can be modulated through therapeutic interventions, even in patients with chronic, severe motor impairments. This aspect provides evidence of cortical adaptability and reinforces the role of non-primary motor areas in supporting motor recovery after stroke (Funato et al., 2022).

Another study by Lai et al. (2016) assessed whether EEG–EMG coherence can detect changes in corticomuscular control resulting from peripheral electrical stimulation. Electrical stimulation (ES) applied to peripheral nerves has been shown to promote brain plasticity and is used in clinical settings to aid motor recovery in individuals with CNS lesions. Despite its use in clinical settings, the clinical effectiveness of ES is still limited due to the difficulty of accurately mimicking natural recruitment patterns (Epstein et al., 2025). In biological systems, neurons are naturally recruited from small to large axons, following Henneman’s size principle (Henneman, 1957, 1985). However, external ES reverses this order by preferentially activating large-diameter axons first. This mismatch between physiological and artificial recruitment hierarchies presents a significant challenge for the clinical effectiveness of ES therapies (for a review, see Epstein et al., 2025). Despite this limitation, ES has been shown to improve motor function through peripheral effects like muscle strengthening and reduced spasticity, as well as through cortical effects. Some studies (Shin et al., 2008; Sasaki et al., 2012; Milosevic et al., 2021) have provided evidence that functional ES therapy promotes cortical reorganization. This neuroplasticity is thought to result from the active participation of the patient, as functional ES therapy requires voluntary movement attempts before stimulation is applied. This timing ensures that descending motor commands from the brain coincide with ascending sensory and antidromic signals generated by stimulation. The repeated convergence of these signals is believed to strengthen synaptic connections (Popovic et al., 2002; Milosevic et al., 2021).

In the study by Lai et al., the ES was delivered in 1-ms rectangular pulses at 100 Hz, with a 20-s on/off cycle. The intensity of the stimulation was set at the highest tolerable level for each participant, avoiding muscle contraction or pain. Before and after ES, participants performed a 20-s steady-hold thumb flexion at 50% of their MVC, during which EEG and EMG data were recorded. The authors found a significant increase in EEG–EMG coherence within the gamma frequency band following electrical stimulation, with increases of 22.1% in healthy participants and 48.6% in stroke survivors. Additionally, force steadiness improved in both groups, as evidenced by a reduction in force fluctuation during steady-hold contraction (−1.7% MVC in healthy participants and −3.9% MVC in stroke survivors). These results demonstrate that EEG–EMG coherence can effectively detect electrical stimulation-induced changes in neuromuscular function. Furthermore, given the association between gamma coherence and sensory encoding, the observed improvements in motor performance may be attributed to the enhanced sensory input and sensorimotor integration induced by the stimulation.

### 4.2 Trunk and lower limb functions in ABI

Kukkar et al. (2024) studied changes in functional coupling between the cortex and lower limb muscles across multiple frequency bands during a challenging balance task in chronic stroke survivors. Eleven stroke patients and nine healthy controls performed a balance task on a computerized platform with and without somatosensory input distortion. This distortion was created by sway-referencing the support surface to vary task difficulty. CMC was calculated between EEG and leg muscles, and balance performance was evaluated using the Berg Balance Scale (BBS), Timed Up and Go (TUG), and COP measures. These authors found reduced delta frequency band coherence in stroke patients compared to healthy controls under medium-difficulty conditions for distal, but not proximal, leg muscles. Both groups exhibited similar coherence levels in other frequency bands. Stroke patients also demonstrated poorer balance on the BBS and TUG, though COP measures did not consistently reflect these group differences. The distal versus proximal muscle effects indicate potential differences in corticospinal reorganization between these muscle groups for balance control. The reduced delta coherence in stroke patients is suggested to stem from altered mechanisms for somatosensory modulation in response to sway-referencing, impacting balance control. Regarding COP measures, the authors found these to be sensitive to variations in task difficulty during balance assessments. However, COP metrics alone were insufficient to distinguish between the postural control strategies employed by stroke participants and healthy controls. In contrast, power spectral density (PSD) analysis, by decomposing the signal into its frequency components, allows for a more detailed examination of the sensory contributions to postural control, identifying subtle balance impairments related to aging and stroke (Kukkar et al., 2024).

Xu et al. (2023) observed stroke patients’ brain control of lower limb movement during ankle dorsiflexion using EEG and EMG to assess muscle control. They recorded EMG from the Tibialis Anterior (TA), Lateral Gastrocnemius (LG), and Medial Gastrocnemius (MG) muscles during a static ankle dorsiflexion task. Stroke patients had significantly lower mean beta and gamma CMC values compared to healthy controls. While healthy controls showed significant coherence in the central cortex, stroke patients did not. The authors suggest that CMC is impaired in stroke patients compared to healthy individuals, indicating reduced connectivity between the brain and lower limb muscles during ankle dorsiflexion. Moreover, they found a significant correlation between CMC and lower limb FMA scores, which implies that higher CMC is associated with better motor function in the lower limbs. In addition, they also found that results from the multiple linear regression model between CMC and lower limb FMA were significant, indicating that CMC could be used to predict lower limb FMA. In this sense, administering a clinical scale such as the FMA may indeed be more feasible, given its strong correlation with CMC. FMA is easier to administer, requiring no specialized equipment or additional cost. However, CMC provides more informative data and represents an assessment that is not solely based on clinical observation. In fact, the CMC is considered a marker of the corticospinal pathway based on the functional coupling between oscillatory signals from the brain and active muscles (Conway et al., 1995). This indicates that CMC may serve as a valuable biomarker for assessing motor control and recovery in stroke patients.

Bao et al. (2021) investigated the directional changes in cortico-muscular interactions after repetitive rehabilitation training with neuromuscular electrical stimulation by measuring EEG and EMG. This kind of rehabilitation treatment has been widely used for motor restoration following a stroke. However, its effects on the closed-loop sensorimotor control process are not well understood. These authors assessed lower limb isometric contractions of both affected and not sides before and after the entire neuromuscular ES session. The analysis focused on CMC and generalized partial directed coherence (GPDC) values between eight selected EEG channels in the central primary motor cortex and the EMG channels. The results showed that rehabilitation training significantly strengthened corticomuscular interactions between the ipsilesional cortex and paretic lower limb muscles. Furthermore, both the descending and ascending cortico-muscular pathways were modified following the training, suggesting that EEG and EMG can be valuable tools for understanding neuromuscular changes during the post-stroke motor rehabilitation process.

Moreover, Short et al. (2020) used EEG during treadmill walking to explore the cortical mechanisms underlying gait in children with and without cerebral palsy (CP). The authors collected lower limb sEMG data and analyzed muscle synergies to assess motor output. EEG data were recorded during a standing baseline and while walking on a treadmill. In particular, they found that the CP group showed greater cortical activation during walking, indicated by increased mu- and beta- event-related desynchronizations in motor and parietal regions, as well as elevated low gamma activity in frontal and parietal areas. Additionally, gamma-band EEG–EMG coherence with the hallucis longus muscle was significantly higher bilaterally in the CP group compared to controls. Overall, the findings suggest that increased cortical activation in children with CP may relate to differences in motor control on the more affected side, highlighting the need for strategies that reduce cortical activation while improving motor control.

Other authors investigated the fatigability in the lower limbs in patients with CP. Particularly, Forman et al. (2022) compared changes in corticospinal drive following sustained muscle contraction in adults with CP and healthy controls. They administered a 1-min static dorsiflexion at 30% of MVC before and after a submaximal contraction at 60% MVC. In addition, they registered EEG and EMG from TA and quantified the coupling expressed as CMC. Patients with CP exhibited lower MVCs compared to their peers but demonstrated similar times to exhaustion during a fatigue trial at a relative load. Both groups experienced changes in EMG median frequency and amplitude related to fatigability. Before experiencing fatigue, the CP group had lower CMC in the beta band; however, both groups showed a decrease in beta band CMC after fatigue was induced. A linear correlation was found between the reduction in beta band CMC and the increase in EMG associated with fatigability. The decrease in beta band CMC after static contraction until failure was linked to fatigability in both healthy adults and adults with CP. The results indicate that both groups rely on compensatory mechanisms to cope with fatigue, with similar influences on corticospinal drive.

## 5 Neurobiomechanical assessment approach in patients affected by neurodegenerative disorders

Neurodegenerative disorders are a heterogeneous group of pathologies that have in common a progression of neuronal loss, neuron structure, and/or their functions (Lamptey et al., 2022). The most common neurodegenerative diseases are Parkinson’s disease (PD), multiple sclerosis (MS), Alzheimer’s disease (AD), spinocerebellar ataxia (SCA) and amyotrophic lateral sclerosis (ALS). However, in this review, we included studies on PD, MS, SCA, and ALS, as shown in Table 2, since these neurodegenerative disorders are typically associated with prominent motor impairments. These disorders tend to cause a constant worsening of postural control, gait functions, and manual abilities, even in the very early stages of the disease. In contrast, AD is primarily characterized by cognitive decline, and motor symptoms, when present, tend to appear only in the later stages of the disease. In this way, some specific features of movement patterns can occur for each pathology, and clinicians should consider them during the rehabilitation process because they could require different types of motor training.

**Table 2:** Summary of the selected studies on PD, MS, SCA and ALS.

First author and year of publication	Function/body target	Study population and sample characteristics	Assessment methodologies	Parameters analyzed	
				Neurophysiological	Biomechanics
Caviness et al. (2006)	Posture/postural tremor.	51 participants, divided into four groups: 14 healthy subjects without postural tremor, 8 healthy subjects with small-amplitude postural tremor (mean age of approximately 79 years) (±9 years for the group without tremor and ±8.9 years for the group with tremor), 10 PD with a mean age of 74.5 years (±10.2) for the group with tremor and 79.1 years (±8.4) for the group without tremor.	EMG, including the deltoid, biceps, triceps, wrist flexors and extensors, abductor pollicis brevis, first dorsal interosseous, and abductor digiti minimi. EEG. Accelerometer (Kistler A250) was applied to the dorsum of the right hand, just proximal to the third metacarpophalangeal joint, to detect movement along the vertical axis.	CMC.	Postural tremor.
Roeder et al. (2020)	Lower limb/gait function.	22 healthy young participants (with a mean age of 25.9 years), 24 old healthy participants (with a mean age of 65.1 years). 20 PD patients (with a mean age of 67.4 years), (50% male in the older and 60% in the PD group). PD participants were in the early stages of the disease (Hoehn & Yahr score 1.3) and had an average MDS-UPDRS score of 38.8.	EEG (32 channels) during walking. EMG recordings from bilateral TA muscles, to measure muscle activity during the phases of the gait cycle. Plantar switches (footswitches) placed under the heel and big toe of both feet were used to identify kinematic events such as heel contact and toe release.	CMC. EEG spectral power. EMG power and EMG amplitude (specific times). Spectral parameters (EEG power, EMG and coherence).	Gait spatial-temporal kinematics.
Yokoyama et al. (2020)	Lower limb/gait function.	34 males with PD, 1) 10 individuals with PD [61.6 ± 6.3 (means ± SD) yr]. 2) 9 age-matched healthy older adults (64.9 ± 6.3 yr). 3) 15 healthy young adults (26.7 ± 7.5 yr).	EEG. EMG from: TA, MG. Optical motion capture system (Vicon Motion Systems, UK) with 9 cameras and retro-reflective markers. Markers positioned according to the Plug-In Gait set to detect kinematic parameters.	CMC. EEG spectral power related to motor control.	Gait spatial-temporal kinematics.
Zokaei et al. (2021)	Upper limb/grip task.	17 PD and 17 healthy controls.	MEG, Elekta Neuromag system with 306 channels. Used to measure cortical neural activity during the grip task. EMG from superficial flexor muscle of the fingers with reference on the lateral epicondyle. Force sensors (Gripper), MEG-compatible devices based on optical fibers.	CMC. Variability in EMG signals (as an indicator of tremor and muscle instability).	Grip Force was measured using MEG-compatible force sensors (grippers). Main parameter: magnitude of force exerted during the gripping task.
De Sanctis et al. (2020)	Lower limb/dual task during walking.	13 RRMS, (10 F and 3 M, with a mean age of 34.9 years), and 15 healthy controls (9 F and 6 M, with a mean age of 34.6 years).	MoBI: EEG system a 64-channel setup (BioSemi ActiveTwo). 3 pressure sensors were placed on each foot at key points: the heel, the base of the big toe, and the longitudinal arch. Kinematic data were acquired using a 3D motion capture system, which allowed for a detailed study of gait dynamics during the execution of the dual task.	The modulation of the N2 wave during the Go/NoGo task was analyzed.	The average walking speed, stride time during the dual task. Kinetic parameters (Forces and Interactions with the Ground) were analyzed using three pressure sensors to monitor the distribution of forces at the following points on the foot: heel, base of the big toe, and longitudinal arch. Specific data on force peaks or other measures of contact forces between the foot and the ground were not detailed in the document.
Tomasevic et al. (2013)	Upper limb/handgrip task.	20 RRMS patients (13 F and 7 M with a mean age of 37.2 years ± 6.1).	23-channel EEG recordings EMG recordings from the right and left opponens pollicis muscles. ECG was also performed to monitor the heart rhythm during the motor task. MRI scanner: 1.5 T (Achieva, Philips Medical Systems). The InPresS pressure device measured the applied force, providing visual feedback to monitor performance.	CMC. Spectral analysis.	Movement accuracy.
Proudfoot et al. (2018)	Upper limb.	ALS (N = 17 with a mean age of 62.6 ± 9.4, 12 M and 5 F) and healthy controls (N = 11, with a mean age of 60.2 ± 12.5, 8 M and 3 F)	MEG data were acquired on an Elekta Neuromag 306 channel scanner at the OHBA. Blinks and saccades were monitored continuously using a combination of surface electro-oculography and infra-red eyetracker. ECG was monitored at the wrists. Surface EMG from flexor digitorum superficialis.	CMC.	Grip strength was recorded via a fiber-optic auxotonic-force response device (resistance increasing linearly with displacement) optimized for use in a scanning environment (Current Designs, USA). Infra-red eyetracker.
Velázquez-Pérez et al. (2017)	Upper limbs/fingers and wrist flexion movements. Lower limbs/foot dorsal extension movements.	19 SCA2 patients (7 M, 12 F, with a mean age 45.21 ± 9.83 years). 24 healthy non-paid volunteers (10 males, 14 F with a mean age 43.83 ± 10.39 years).	EEG. EMG from FDS muscles of the right upper limb and in the TA of the right lower limb. Manual digital dynamometer used to measure the level of MVC during motor tasks of the upper limbs. TMS used to determine CMCT in the muscles of the hand and right leg, assessing the integrity of the corticospinal tract.	CMC. CMC inversely correlated with CMCT. EMG activity during isometric muscle contractions at 30% of the MVC level.	MVC during motor tasks of the upper limbs.

The table reports the characteristics of the included sample, target function, technological equipment for the neurobiomechanical assessment, and neurophysiological and biomechanical parameters.

M (Male), F (Female), Electroencephalography (EEG); Electromyography (EMG); CMC cortico-muscular coherence, magnetencephalography (MEG), Parkinson’s disease (PD), Unified Parkinson’s Disease Rating Scale (UPDRS), MS (multiple sclerosis); Co-contraction Index (CI); electrical stimulation (ES); Fugl-Meyer (FMA); healthy controls (HC); Plantar switches (footswitches); typical development (TD); Magnetic resonance imaging (MRI); Spinocerebellar ataxia type 2 (SCA2); Transcranial Magnetic Stimulation (TMS); central motor conduction time (CMCT); Direct cortico-muscular coherence (dCMC); transcranial magnetic stimulation (TMS); somatosensory evoked potentials (SEPs); Electro-oculography (EOG); Tibialis Anterior (TA); Gastrocnemius medialis (GM); Mobile Brain/Body Imaging (MoBI); Relapsing remitting multiple sclerosis (RRMS); InPresS (Interactive Pressure Sensor); Electrocardiogram (ECG); Oxford Center for Human Brain Activity (OHBA); flexor digitorum superficialis (FDS); Maximum voluntary contraction (MVC).

### 5.1 Upper limb functions in neurodegenerative diseases

Zokaei et al. (2021) studied the changes in CMC in individuals with PD, comparing them to healthy controls with matched grip strength. The PD group showed a significant reduction in beta CMC, despite having similar grip strength to controls. In addition, PD patients showed significant reductions in power in the alpha (6–8 Hz) and beta (15.5–37 Hz) bands compared to healthy controls. The spatial distribution of CMC revealed that the coupling between cortex and muscles was localized to specific motor regions, but the magnitude of coherence was lower in patients with PD. Despite the reduction in CMC in the beta frequency, PD patients showed force stability in the grasping task. However, this result may be attributed to greater variability in EMG signals, likely reflecting tremor-related fluctuations in muscle activity.

In patients affected by MS, fatigue is a prevalent symptom that severely affects the quality of life. Previous research indicating altered connectivity patterns suggested that disruptions within the brain-muscle circuit may play an essential pathogenic role in MS-related fatigue. In the study of Tomasevic et al. (2013), the authors used structural measures (MRI to assess thalamic volume and cortical thickness in primary sensorimotor areas) and functional measures, such as CMC, from simultaneous EEG and sEMG recordings during a light handgrip task. CMC in the beta band (16–30 Hz), fatigued patients showed higher frequency CMC than non-fatigued patients, indicative of an increase in functional communication between the primary motor cortex (M1) and muscles during the motor task. The frequency of CMC explained 67% of the variance in the Modified Fatigue Impact Scale (MFIS) scores, making it a sensitive marker of fatigue. The changes observed in the CMC between non-fatigued and fatigued patients suggest an alteration in the quality of the functional connection, rather than in the power of isolated brain activity. Functional communication between the motor cortex and muscle was impaired in fatigued patients, highlighting an overload of the cortical system to maintain motor performance similar to non-fatigued patients. The differences in CMC were independent of EEG or EMG amplitude, suggesting that the impairment lies in the quality of the functional connection between brain and muscle. The accuracy of the hand grip task was comparable between fatigued and non-fatigued patients, despite differences in correction frequency.

Other authors (Proudfoot et al., 2018) investigated the use of CMC to assess oscillatory interactions between cortical areas and muscles during motor tasks in primary lateral sclerosis, which is a neurodegenerative disorder of upper and lower motoneurons, as well as to explore the relationship between CMC and clinical impairment levels. In particular, they recorded EEG and EMG data from hand muscles while performing a pincer grip task. They found that CMC was elevated over contralateral-M1 (in alpha- and gamma-bands) and ipsilateral-M1 (in the beta-band) compared to controls. Correlation analyses showed that greater clinical impairment was linked to lower CMC in the contralateral-M1/frontal areas, higher CMC in the parietal region, and mixed CMC levels (both increased and decreased) in different frequency bands over ipsilateral-M1. These findings suggest unusual involvement of both contralateral and ipsilateral M1 during motor tasks in primary lateral sclerosis, pointing to possible harmful or compensatory changes in brain activity. The findings underscore the potential of CMC as a marker for identifying sensorimotor network dysfunction in this patient population.

### 5.2 Trunk and lower limb in neurodegenerative disease

Changes in human gait due to aging or neurodegenerative diseases are influenced by multiple factors. For example, Roeder et al. (2020) evaluated the impact of age and PD on corticospinal activity during treadmill and overground walking. EEG data were recorded from 10 electrodes, and EMG signals were acquired from bilateral TA muscles. Event-related power, CMC, and inter-trial coherence were analyzed for EEG data from bilateral sensorimotor cortices and EMG during the double-support phase of the gait cycle. The results indicated a significant reduction in CMC and EMG power at low beta frequencies (13–21 Hz) in older adults and those with PD compared to younger individuals, with no significant difference between the older and PD groups. Additionally, both older and PD participants spent less time in the swing phase of gait compared to young adults. These findings suggest that aging affects the temporal coordination of gait. The observed decrease in low-beta CMC implies diminished cortical input to spinal motor neurons in older individuals during the double-support phase. These authors also noted various changes in electrophysiological measures at low-gamma frequencies between treadmill and overground walking, indicating task-dependent differences in corticospinal locomotor control. Spectral parameters (EEG power, EMG and coherence) were generally reduced during treadmill walking compared to walking on natural terrain, suggesting a different neurophysiological control between the two walking modes. Regarding biomechanical findings, older PD patients showed reduced time in the swing phase single support compared to young participants, while the duration of the double support time was similar between the two groups. EMG power showed an overall reduction in elderly and Parkinson’s subjects, suggesting changes in muscle activation and motor modulation during the gait cycle.

Similarly, Yokoyama et al. (2020) assessed the influence of PD and aging on CMC during walking by recording EEG and EMG signals. They measured CMC between the motor cortex and two lower leg muscles, TA and MG, during walking. Healthy controls (both older and younger groups) showed distinct muscle activation peaks at specific gait phases, while the PD group displayed prolonged activation patterns. CMC was lower in the PD group than in healthy older adults in the alpha-band (8–12 Hz) for both muscles and in the beta-band (16–32 Hz) for the TA. The reduced CMC suggests impaired cortico-muscular communication, likely resulting from dysfunctions in motor control and sensory integration processes. Patients with PD showed a marked reduction in CMC in the alpha band, indicating a possible deficit in sensory feedback management during walking. The elderly had CMC similar to the young, suggesting that the deficit observed in PD patients was disease-specific and not age-related. In particular, CMC was analyzed during different phases of the gait cycle: initial load and foot strike and during the terminal stance, and pre-swing. In PD patients, the EMG peaks registered during the gait analysis were attenuated, reflecting a weaker synchronization between the motor cortex and muscles. The direction of the CMC was predominantly downward (from cortex to muscles) in the alpha and beta bands. In Parkinson’s patients, this preferential direction was absent, indicating a loss of effective cortical motor control. These alterations detected at the neurophysiological level (EEG–EMG) also have repercussions at the biomechanical level. For example, these authors found that patients with PD had a significantly shorter stride length than healthy controls. The stride time showed no significant differences between the groups, indicating that the patients compensated for the reduced stride length by maintaining a similar cadence.

Other studies analyzed the CMC in PD patients to assess neuromuscular patterns of tremor. Nowadays, the exact mechanisms and electrophysiological features of postural tremor in PD remain unclear. It was hypothesized that individuals with PD who exhibit small amplitude postural tremor would demonstrate heightened CMC at specific frequencies compared to those without visible tremor. In the study of Caviness et al. (2006), four participant groups were examined: (1) Controls without postural tremor, (2) Controls with small amplitude postural tremor, (3) PD patients without postural tremor, and (4) PD patients with small amplitude postural tremor. The authors recorded data from accelerometers along with EEG–EMG fast-Fourier transform and CMC spectra. The frequency band between 5 and 8 Hz was associated with the mechanical components of the tremor, reflecting muscle oscillations related to the physical movements of the tremor itself. The 8–12 Hz range is particularly relevant for tremors associated with PD, indicating specific brain activity related to the condition. Another band of significant interest is 12–18 Hz, which showed a marked increase in CMC in patients with small-amplitude postural tremor, suggesting greater involvement of the sensorimotor cortex in generating the tremor. Finally, the 18–30 Hz band was used to examine cortical activity during motor tasks, allowing for a better understanding of how the brain manages the control of muscle movements. The observed increase in corticomuscular coupling highlights cortical involvement in PD’s small amplitude postural tremor.

On the other hand, patients with spinocerebellar ataxia type 2 (SCA2) often manifest corticospinal tract dysfunction. In the study of Velázquez-Pérez et al. (2017), corticospinal tract dysfunction in SCA2 patients was assessed with CMC as a measurement tool. In SCA2 patients, CMC was significantly reduced in the lower limbs (TA muscle), but not in the upper limbs. Interestingly, reduction of CMC was also present in patients without clinical signs of corticospinal tract dysfunction. An inverse correlation emerged between CMC for the lower limbs and central motor conduction times to the TA muscle. Lower CMC is associated with longer central motor conduction time, indicating a link between corticospinal tract degeneration and reduced corticomuscular synchronization. The EEG showed that oscillations in the beta band (15–30 Hz) were significantly less synchronized with EMG signals in patients than in healthy controls. On the other side, EMG showed electrical activity during isometric muscle contractions at 30% of the maximum voluntary level.

Other authors studied the neural mechanisms underlying dual-task walking in patients with MS (De Sanctis et al., 2020). They recorded event-related potentials (ERPs) in individuals with MS and healthy controls during a Go/NoGo task, either while seated (single task) or walking (dual-task). The study assessed how brain response modulation related to task demands impacted performance. The authors found that in the Go/NoGo task, individuals with MS experienced a performance decline during dual-task walking, whereas healthy controls exhibited a performance improvement. Additionally, healthy controls showed task load-dependent modulation of brain responses, a pattern not observed in the MS group. Analysis of the combined sample indicated a positive correlation between ERP changes associated with task load and dual-task performance. In this way, the results provided by the authors could aid in identifying objective brain measures for real-world challenges, potentially enhancing MS assessment approaches.

## 6 Computational simulation approaches in neurobiomechanical assessment

Recent literature highlights the emerging role of computational strategies in advancing neurobiomechanical assessment and rehabilitation. Instead of providing an exhaustive list of simulation platforms, recent studies focus on methodological innovations and their translational potential. These computational approaches aim to bridge the gap between neurophysiological understanding and clinical application by integrating neural control models, biomechanical analysis, and patient-specific data.

De Groote and Falisse (2021) provide a foundational perspective on musculoskeletal modeling for predictive simulations of human gait. Their work illustrates how simulations can elucidate altered movement patterns associated with neurological disorders and offers a framework to assess therapeutic strategies in silico. In the domain of neurodegenerative disease, Elias et al. (2018) explore how neuromusculoskeletal modeling helps investigate disruptions in sensorimotor control, focusing on the interaction between cortical command and muscular execution.

Innovative signal processing methods also contribute to computational neurobiomechanics. Borzelli et al. (2025) introduce the pooled scalogram, a wavelet-based technique designed to detect muscle co-activation patterns in the time-frequency domain, which may be instrumental in characterizing compensatory strategies in motor-impaired patients.

Wearable and real-time modeling technologies further extend clinical applicability. Simonetti et al. (2022) integrate rapid musculoskeletal modeling with smart garments, enabling dynamic assessments that support neuromechanical decision-making in rehabilitation contexts. Similarly, Garro et al. (2021) advocate for the identification of neuromechanical biomarkers to guide robotic neurorehabilitation interventions.

Direct clinical applications are also documented. Romanato et al. (2022) employed biomechanical modeling in a randomized trial to assess the impact of exoskeleton training in Parkinson’s disease, providing an example of simulation tools used for outcome quantification in a clinical setting. Gogeascoechea et al. (2020) show how modeling neural interfaces can optimize stimulation parameters in spinal cord injury rehabilitation.

Additional evidence from Norton and Gorassini (2006) demonstrates that changes in IMC reflect functional motor improvements in incomplete spinal cord injury, reinforcing the value of computational coherence analysis. Finally, Kizyte (2025) presents a high-density EMG-informed framework for assessing muscle control integrity, offering a promising path toward individualized biomechanical modeling.

## 7 Discussion

This review comprehensively explored the neurobiomechanical assessment in specific neurological conditions, including acquired brain injury (e.g., stroke, cerebral palsy) and neurodegenerative disorders (e.g., MS, PD, and SCA). The integration of neurophysiological tools, such as EEG and EMG, with biomechanical assessment provides valuable insights into movement, spanning from cortical activity to muscular and joint function. Combining multiple sources of information can uncover specific causes of impairment that may not be evident through clinical scales alone. Although techniques such as EMG, EEG, and MoCaps are not yet widely adopted in clinical settings, multi-parameter assessments show promise in detecting motor improvements that could be overlooked when clinical evaluations are limited to a single domain (Scano et al., 2023). A potential limitation of clinical scales is that they may be too qualitative and might miss specific patients’ needs. For example, single-item scales, though easy for clinicians to understand and communicate (e.g., Expanded Disability Status Scale – EDSS scores), are scientifically limited (Hobart et al., 2007). These tools often lack reliability, validity, and responsiveness due to random error, ambiguity, and overly broad scoring categories (Hobart et al., 2007). In contrast, multiple-item scales (e.g., Fugl-Meyer) combine responses from several items, which improves reliability (by reducing random error), validity, and responsiveness by allowing a more granular and accurate representation of complex variables (Hobart et al., 2007). However, such scales may still fail to capture movement quality and are often insensitive to distinguishing whether improvements result from true motor recovery or compensatory strategies (Kwakkel et al., 2019). As proposed by Kwakkel et al. (2017), the use of technologies that enable objective measurement of movement kinematics and kinetics represents the most promising approach to address this limitation. In this sense, future studies could integrate a neurobiomechanical assessment as a potential approach for individualized assessment, registering the specific neurophysiological and biomechanical activity. On the other hand, clinical scales often do not allow this degree of personalization, and they are difficult to adapt to each patient. Nonetheless, clinical scales retain practical advantages: they require no specialized equipment like MoCap or EMG/EEG systems, making them more accessible and cost-effective for routine clinical use. However, a multi-domain assessment approach could offer a more comprehensive understanding of the mechanisms underlying the relearning process and the specific level (neural or muscular) at which it occurs after rehabilitation.

In this review, we tried to answer three questions: “Which technologies can perform a neurobiomechanical assessment in neurological patients?,” “What are the key neurophysiological and biomechanical parameters?,” “How can we translate this approach from research to clinical practice?”

### 7.1 Which technologies can perform a neurobiomechanical assessment in neurological patients?

The collected evidence suggests that neurobiomechanical assessment does not depend on a single instrument but rather on a combination of systems (e.g., EEG, EMG, MoCap, WS, and force sensors) that collectively capture distinct aspects of human movement. In particular, the most commonly used technologies in the neurophysiological field include 64-channel EEG and surface EMG, while biomechanical assessments often incorporate force sensors to measure grip strength and MVC, along with MoCap systems for detailed kinematic analysis.

Additionally, some researchers have incorporated neuroimaging techniques, such as MRI, with neurophysiological methods (e.g., MEG/EEG) to further enhance the assessment of the complexity of human movements in patients affected by neurological disorders.

Each tool, device, or sensor used alone provides valuable information within its specific domain, but provides only a partial view of the patient’s motor function. A more comprehensive patient assessment, which considers multiple aspects of movements (e.g., neural and muscle activity, biomechanics), is essential for understanding the peculiar features of movement, as each pathology affects individuals differently. Multi-domain approaches enable the evaluation of neuromotor organization at various levels, facilitating personalized therapy adjustments (Scano et al., 2023). In this context, integrating MoBI technologies enables assessments during dynamic, real-world tasks, offering insights into multitasking and postural control in natural settings (Delaux et al., 2021). De Sanctis et al. demonstrated the usefulness of this approach for identifying EEG-based neuromarkers during a dual-task paradigm, where individuals with MS walked while performing a concurrent cognitive task (Go/NoGo). It is worth noting that neuroimaging methods (e.g., fMRI) can be combined with MoCap systems to obtain further valuable insights into the understanding of human movement control as well as pathological mechanisms (e.g., bradykinesia) (Sarasso et al., 2024). However, this approach has some limitations, such as the physical restrictions inside the scanner (e.g., lying down, limited space), which alter natural movement patterns; movements inside the MRI may not reflect real-life or outside-the-scanner performance. In addition, another key limitation of combining fMRI with kinematics is the mismatch in sampling rates, while MoCap systems record data at high frequencies (e.g., 60 Hz), fMRI captures brain activity more slowly (every 1–3 s), making precise alignment difficult and risking loss of movement detail. To integrate the two, kinematic data are often down-sampled, which can distort repetitive or fast motions and misrepresent motor activity (Belkacemi et al., 2025).

In other studies (Fang et al., 2009; Belardinelli et al., 2017; Guo et al., 2020; Zhou et al., 2021), multi-parameter assessments have been performed by robotic devices used in rehabilitation settings. These devices are equipped with sensors, measuring biomechanical parameters such as kinematics and force data, enhancing objectivity, repeatability, precision, and ease of use. This integration allows for real-time adaptation of rehabilitation strategies based on motor performance, which can be promptly and accurately analyzed by embedded sensors (Bonanno and Calabrò, 2023). This approach has been primarily applied to the upper limb in neurological patients (Fang et al., 2009; Belardinelli et al., 2017; Guo et al., 2020; Zhou et al., 2021). However, it could also be extended to the lower limb using devices like the Lokomat, as demonstrated by Artoni et al. (2023) in healthy subjects. In this sense, future studies should integrate robotic devices, such as Lokomat, for neurobiomechanical assessment of gait functions, especially for patients with severe impairments where gross motor movements are not easily observable. A key consideration is that robotic devices, such as Lokomat, often involve lengthy setup procedures and typically require the assistance of technical personnel. In clinical environments, physiotherapists usually have only 30–60 min per session per patient, which may further limit the practicality of integrating robotics into routine care. Moreover, robotic systems, particularly exoskeletons, need to be precisely aligned with the user’s joints to prevent the generation of unintended interaction forces. If these forces become excessive, they can compromise both comfort and safety during use. To mitigate joint misalignment, soft exoskeletons made of flexible textiles or elastomers have been proposed, as they offer improved adaptability and comfort compared to rigid robotic orthoses. Despite their potential, robotic devices remain inaccessible in many rehabilitation centers due to high costs, ongoing maintenance demands, and the requirement for specialized staff. These factors likely contribute to the limited adoption of robot-based assessments in everyday clinical practice (Bonanno and Calabrò, 2023).

Despite these limitations, a previous study (Calabrò et al., 2017) has proven that EEG during Lokomat training sessions is feasible for post-stroke patients, finding significant brain activation (mu/beta event-related spectral perturbations - ERSPs) during robotic therapy plus virtual reality. Another advantage of using multidomain instrumental evaluation is that it allows for a deeper understanding of the mechanisms underlying clinical improvements observed in patients. By analyzing neural, biomechanical, and physiological changes, this approach helps identify the specific processes contributing to motor recovery, enabling more targeted and effective rehabilitation strategies.

Despite these advantages, multi-domain instrumental approaches face several limitations that prevent their widespread use in clinical practice. One major disadvantage is the need for patients to wear multiple systems simultaneously (e.g., EEG headsets, surface EMG electrodes, and motion capture devices, both MoCap and WS), which can restrict natural movement expression and compromise assessment accuracy. Additionally, the setup requires significant preparation time, making it impractical for routine clinical assessments where time is limited. The high cost of these technologies further challenges their accessibility to many facilities. Moreover, the use of multiple sensors and cables can lead to technical issues such as interference, sensor displacement, and motion artifacts potentially affecting data reliability. In addition, another limitation of using multiple systems simultaneously is the need for device integration and synchronization among acquiring systems, which are not always provided as an option by manufacturers and can be challenging to implement without specialized engineering expertise. From a patient perspective, wearing numerous devices may cause discomfort, influence psychological state, and alter movement performance, reducing the overall transparency and effectiveness of the assessment (Scano et al., 2023). In future studies, it is essential not only to develop compact and user-friendly tools but also to ensure that these tools facilitate synchronization processes across different devices. These new solutions should be designed to enhance efficiency, accessibility, and sustainability while addressing the limitations identified in the selected evidence discussed in this review. By integrating innovative approaches, these tools can contribute to reducing resource consumption, optimizing performance, and facilitating broader adoption across various applications.

### 7.2 What are the key neurophysiological and biomechanical parameters?

According to the selected evidence, CMC can be considered a key parameter for understanding the functional connection between the brain and muscles. Through EEG and EMG techniques, CMC has emerged as a tool to assess this interaction by examining neural oscillations in the cortex and muscles. Motor impairments in stroke patients are primarily caused by disrupted transmission of neural oscillations, which compromises connectivity along the corticospinal pathway and weakens cortical control over muscle function (Wang and Choi, 2020). On the other hand, biomechanical spatial-temporal parameters (e.g., stride time, cadence, speed, stride length) to assess gait functions as well as MVC for grip force were commonly extracted from the selected evidence. In addition, the measurement of torque was considered crucial by some authors (Forman et al., 2022) for assessing muscle capacity (MVC) and fatigability during the 60% MVC contraction test. Torque provides a direct indication of muscle capacity and the effect of fatigue on performance. Regarding the biomechanics of upper limb movements, several studies have highlighted alterations in kinematic parameters such as angular velocity, movement smoothness, and joint coordination in individuals with neurological disorders, particularly post-stroke. For instance, Hogan and Krebs (2004) developed robotic assessments to quantify upper limb impairment through kinematic indices, such as smoothness and speed, offering objective measures of motor recovery. Similarly, Krakauer et al. (2000) demonstrated that early post-stroke arm reaching is characterized by decreased peak velocity and increased movement variability. Lin et al. (2019) analyzed velocity profiles and joint trajectories to assess motor planning deficits in stroke survivors. From a motor control perspective, d’Avella and Bizzi (2005) and Santello et al. (2013) investigated the modular organization of movement, proposing that impaired synergies may underlie dysfunctional coordination post-lesion. Furthermore, Raghavan (2015) emphasized the role of abnormal joint torque coupling and reduced inter-joint coordination in upper limb hemiparesis.

Other authors (Krauth et al., 2019; Xu et al., 2023) used clinical scales in addition to neurophysiological evaluation. Although clinical scales, such as Fugl-Meyer, may be useful to assess motor impairment and recovery, they are not able to measure brain-muscle interaction. For instance, a significant CMC may indicate corticospinal pathway use, while a lack of CMC may suggest diverse neural oscillation strategies rather than corticospinal pathway non-utilization. CMC is thus valuable for studying abnormal neural transmission in stroke patients, potentially pointing out the progression of patients over time, during rehabilitation (Gao et al., 2024). In this sense, the beta band was particularly highlighted in the selected evidence, as it is strongly associated with motor control and neuroplasticity. However, in the study by Xu et al., the authors found a correlation between FMA scores and CMC in post-stroke patients, suggesting that higher CMC values were associated with better motor performance as measured by the FMA. In this vein, one might argue that administering a clinical scale could be a more practical and less resource-intensive option than using an EEG–EMG setup. Nevertheless, it is important to note that clinical scales like the FMA may not be sufficient to distinguish true behavioral restitution from compensatory strategies following stroke. Brain–muscle functional connectivity, as measured by EEG, shows changes in beta and low gamma band activity, frequencies associated with motor control, suggesting neuroplastic adaptations during rehabilitation. Analyzing the power density of these cortical rhythms offers new insights into the understanding and management of neurological diseases (Bao et al., 2021). The beta band may reflect the involvement of mechanisms controlling voluntary motor activity through the corticospinal system and appears to undergo modifications after a stroke (Delcamp et al., 2023). A study conducted over post-stroke patients reveals that event-related desynchronization in the beta band was highest among bilateral sensorimotor areas, during elbow extension movement. As a result, the higher CMC during the acceleration phase suggests the loss of motor command following stroke (Fauvet et al., 2021). From a biomechanical perspective, post-stroke patients showed a significant reduction in elbow range of motion and peak angular velocity compared to healthy individuals. This suggests that post-stroke patients move more slowly with less amplitude of movements. Additionally, movement smoothness was impaired, particularly during the deceleration phase, with a significant increase in acceleration peaks, indicating less fluid motion.

Interestingly, in another study conducted by Krauth and colleagues, the authors reported that CMC changed over time. Patients were instructed to do wrist extension during three sessions, accompanied by rest, monitored by EEG-64 channels and EMG. During these movements, it was highlighted that the peak of beta coherence increased over ipsilateral and contralateral M1 during the third session compared to the first and second. Meanwhile, coherence between the first and the second session has low significance. On the other hand, healthy subjects, performing movements only in one session, showed EEG–EMG coherence in the beta band localized over M1 across the contralateral hemisphere (Krauth et al., 2019). These results exhibit how motor neurons of cortical areas near the lesioned regions may compensate for motor function losses. The presence of the beta band is associated with submaximal, isometric contractions, although it has also been observed in patients recovering from stroke involving dynamic muscle contraction (Krauth et al., 2019). Some studies (Fang et al., 2009; Tomasevic et al., 2013; Lai et al., 2016; Roeder et al., 2020; Yokoyama et al., 2020; Forman et al., 2022) also explore the gamma band, revealing its role in sensory integration and motor planning, especially post-intervention. For example, Lai and colleagues found that applying peripheral ES to the paretic upper limb enhanced cortico-spinal coherence, indicating neuroplastic changes in the sensorimotor control loop. They also observed an increase in gamma band activity, which is associated with sensorimotor integration, motor planning, cognition, and attention (Lai et al., 2016).

Fatigability was also explored in patients with CP, showing a linear correlation between a decrease in beta-band CMC (Forman et al., 2022). On the other hand, the alpha and gamma bands showed less relevant correlations with motor tasks and fatigue-induced changes. In another study, event-related desynchronizations in the beta and mu (8–13 Hz) bands in CP children reported that CMC was greater on motor and parietal regions (Short et al., 2020). This is because the beta band is closely associated with the motor control of static and submaximal contractions and was sensitive to fatigue-induced changes in both groups (CP and neurologically intact individuals). Based on neurophysiological results, the advantage of matching EEG–EMG is that it allows for better understanding of the connection between neurons in a particular brain region and different muscular districts, with real-time measurement, and it can predict motor recovery (Krauth et al., 2019; Xu et al., 2023).

Regarding neurophysiological parameters in neurodegenerative disorders, the beta-band was generally softened when compared to healthy controls, because of neurophysiological alterations. Interestingly, in the study by Zokaei et al., a reduced CMC in the beta band was observed in individuals with PD. However, this reduction did not appear to significantly impair their ability to perform a grip task. This raises a critical question: what is the true functional role of CMC if motor performance remains preserved despite its reduction? In this study, the authors acknowledge this ambiguity and correctly avoid overinterpreting causality. However, future research should aim to dissect whether CMC reflects a causal mechanism of motor control or is merely an epiphenomenon of altered cortical communication.

Whereas in small postural tremor, the low-frequency beta-band significantly increases, explaining the involvement of the sensorimotor cortex (Caviness et al., 2006). It is worth noting that in the study by Caviness et al., the authors support the heterogeneity of the pathophysiological mechanisms underlying tremor in PD, ranging from central oscillatory processes to peripheral mechanical reflex components. In this sense, CMC could give novel insights into the understanding of specific motor manifestations (e.g., postural tremor) in people with PD.

During walking, other specific neurophysiological parameters were found. Although the CMC decreased during walking in people with PD, it was not influenced by aging. Conversely, the reduction in the alpha band suggests that motor functions are less modulated and more desynchronized (Yokoyama et al., 2020). In particular, the marked reduction in alpha band CMC, which is closely associated with sensory feedback processing, may indicate impaired sensorimotor integration in PD. This aspect aligns with known sensory and proprioceptive deficits in PD patients. Interestingly, unlike in the studies by Zokaei et al. and Yokoyama et al., no correlation was found between CMC and UPDRS scores, which may be due to the bilateral nature of gait and the high individual variability in CMC. Notably, while Zokaei et al. reported that CMC was related to motor symptoms (as measured by UPDRS-III), Yokoyama et al. did not find such an association during gait. This discrepancy might be explained by the bilateral and automatic nature of walking, which may reduce the impact of lateralized symptoms typically captured by UPDRS.

Another study found electrophysiological differences associated with gait measures. Specifically, a decrease in low-beta CMC suggests a reduction in cortical input to spinal motor neurons, which plays a crucial role in controlling gait. Moreover, multiple electrophysiological changes were observed at low-gamma frequencies (30–45 Hz) during treadmill compared to overground walking, suggesting task-dependent differences in corticospinal control (Roeder et al., 2020). These findings indicate that variations in cortical communication may play a role in walking difficulties, particularly in conditions affecting motor control (Roeder et al., 2020). Both studies (Roeder et al., 2020; Yokoyama et al., 2020) accompanied the neurophysiological evaluation with a biomechanical gait assessment, extrapolating spatial-temporal gait features. In particular, these authors noticed a reduced swing phase and single support time in both elderly individuals and people with PD compared to young individuals. In addition, stride time showed no significant differences between groups, indicating compensatory mechanisms in PD patients to maintain cadence despite reduced stride length. These findings were also shown by De Pasquale et al. in PD patients with higher levels of perceived fear of falls. In particular, factors such as fear of falling may influence muscle co-contraction, potentially contributing to the development of compensatory muscle patterns, for example, increased proximal muscle activity in response to reduced distal muscle function (De Pasquale et al., 2025).

Furthermore, a feature that could influence the magnitude of CMC is fatigue. Regarding this one, it was highlighted that comparing CMC between fatigued patients and non-fatigued beta band increased with the result that CMC worked at higher frequencies as fatigue increased in individuals with MS (Tomasevic et al., 2013). From a biomechanical perspective, fatigued patients showed a higher frequency of pressure correction during the gripping task, indicating a greater need for adjustments to maintain the required level (5% of MVC). Regarding movement accuracy, it was comparable between fatigued and non-fatigued patients, despite the differences in correction frequency. Moreover, CMC has also been evaluated in SCA2, demonstrating a cortico-muscular dysfunction in the lower limbs related to a less synchronization of the beta band since the central motor conduction is impaired (Velázquez-Pérez et al., 2017). These parameters are complemented by biomechanical measures like force deviation, co-contraction indices, and kinematic data, which provide critical insights into motor performance and recovery.

### 7.3 How can we translate this approach from research to clinical practice?

**Figure 1:**
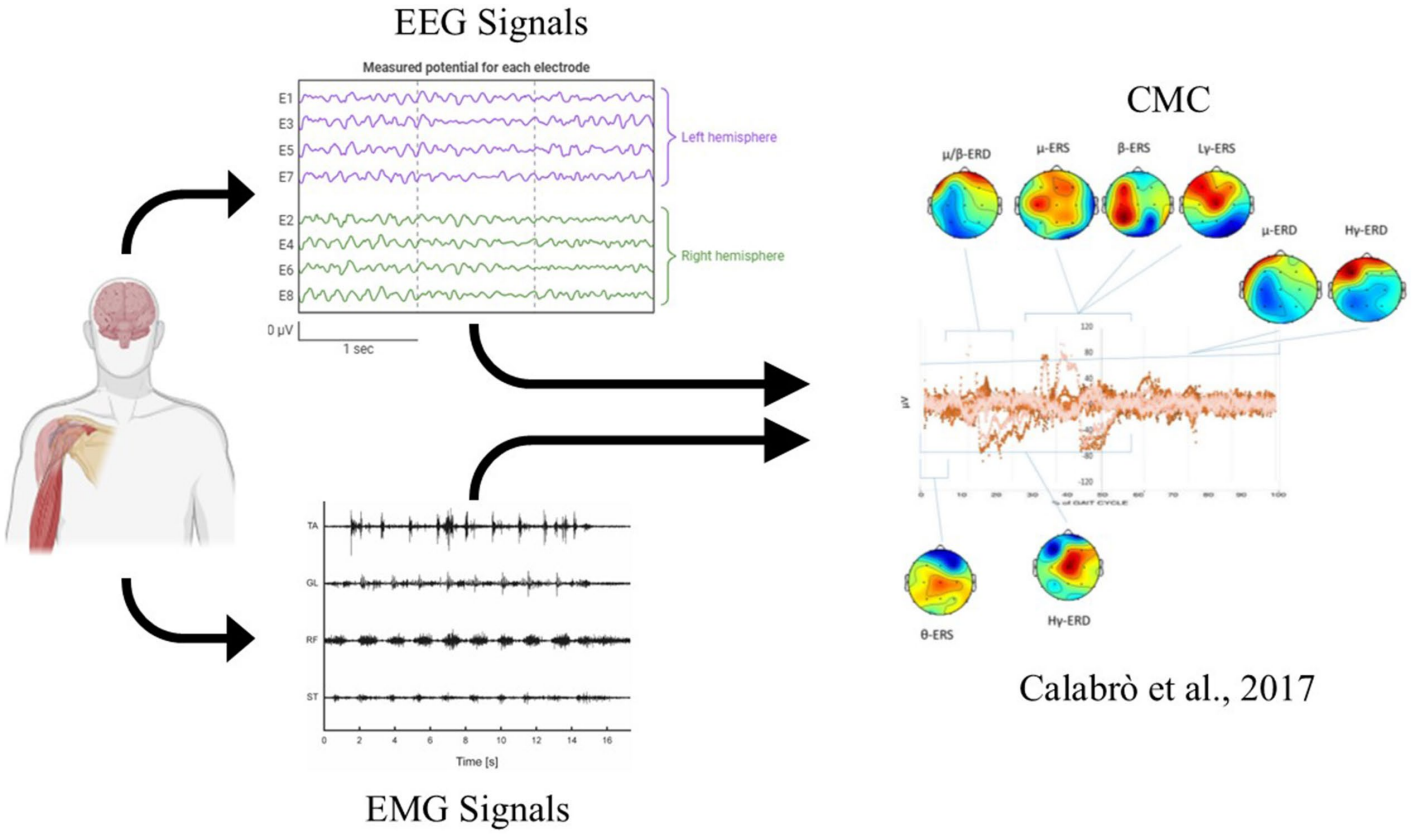
The figure illustrates the combined analysis of EEG and EMG signals, providing a comprehensive view of the motor pathway quantified by CMC. EEG, electroencephalography; EMG, electromyography; CMC, corticomuscular coherence; TA, tibialis anterior; GL, gastrocnemius lateralis; RF, rectus femoris; ST, semitendinosus. The coherence maps are taken from Figure 5 of Calabrò et al. (2017).

One of the primary goals of rehabilitation is to maximize the use of remaining functional abilities. This can be achieved by analyzing cortical pathways (EEG–EMG) to understand the neural control of movement, as well as functional motor activity (EMG-movement) to evaluate how muscle activation translates into movement execution. Examining the entire motor pathway, from cortical activity to final movement execution, might provide crucial insights into individual patient conditions (Figure 1).

**Figure 2:**
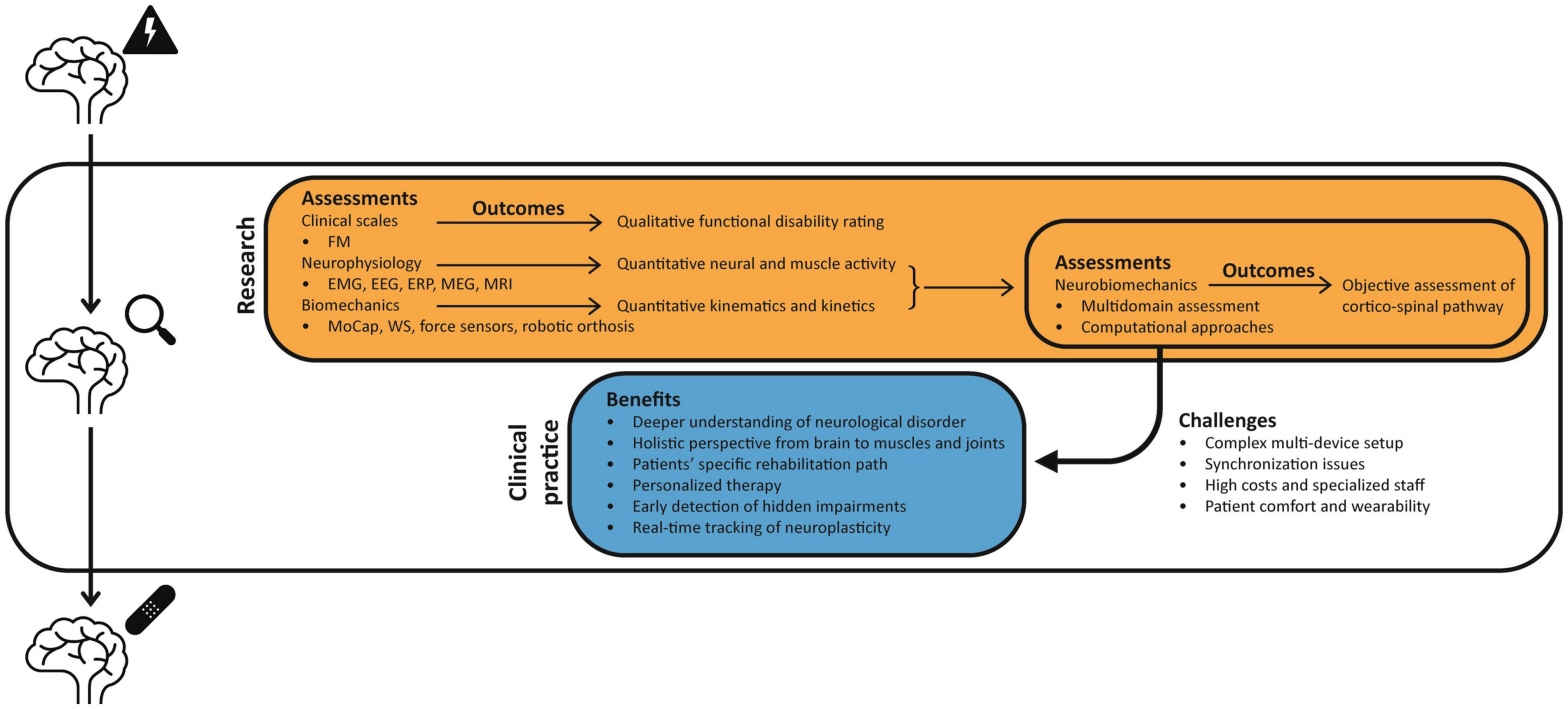
Schematic representation of the implementation of multidomain assessment from research to clinical practice, reporting the challenges and benefits of this assessment approach in the context of neurorehabilitation. On the left, a schematic representation illustrates the patient’s medical pathway from the onset of the neurological disease to a comprehensive motor evaluation, supporting a deeper understanding of the condition and enabling a more tailored rehabilitation approach. On the right, the orange section highlights the main devices (EEG, EMG, MEG, MRI, ERPs) and outcomes (e.g., clinical scales) used in research settings for neurobiomechanical assessment (neurophysiological and biomechanical parameters). The blue block summarizes the benefits of this approach, as well as the challenges that may hinder its implementation in current clinical practice.

This approach enables the creation of personalized rehabilitation protocols tailored to the specific pathology and the patient’s residual capabilities. By integrating neurophysiological (EEG–EMG) and biomechanical data, a more comprehensive and multidimensional assessment becomes possible. However, the greater complexity of experimental setups and the need for synchronization across devices introduce several practical and analytical challenges, including the cost of technology, the need for specialized personnel, difficulties in interpreting results, and the potential for movement alterations caused by multiple wearable devices. Therefore, an optimal balance between data richness and feasibility should be achieved. It is noteworthy that while clinical scales remain an essential tool in neurological assessment, using them in isolation may result in inaccurate evaluations. For instance, Bloem et al. identified several clinical scales that can adequately assess gait and balance alterations in patients with PD. However, they reported that no existing instrument comprehensively and separately evaluates all relevant PD-specific gait characteristics with strong clinimetric properties, nor does any scale provide distinct scores for gait and balance with adequate content validity specific to PD. Therefore, they recommend the development of a comprehensive clinical scale capable of evaluating both aspects independently. This would be preferable instead of relying on multiple existing tools, which may not fully capture the complexity of gait and balance impairments in PD (Bloem et al., 2016). In cases where clinical scales suggest a non-pathological condition, multidomain assessments may reveal hidden motor impairments. For example, mild neurological deficits that are not evident through basic observation or single domain assessment may become apparent when analyzing EEG–EMG patterns and detailed movement biomechanics. Early detection of these issues allows for timely intervention, preventing further deterioration and optimizing rehabilitation outcomes. On the other hand, for patients with severe neurological conditions, standard clinical evaluations may fail to capture subtle motor activity. A simple observation may only determine whether movement is present or absent, without explaining the underlying mechanisms. By integrating neurophysiological and biomechanical assessments, clinicians can better identify whether movement limitations arise from cortical dysfunction, impaired neuromuscular transmission, or biomechanical constraints. From a clinical standpoint, these findings highlight the need for targeted interventions to enhance brain-muscle connectivity (Figure 2).

The translation of neurobiomechanics from research to clinical practice depends not only on experimental validation but increasingly on the integration of computational modeling and simulation tools. Platforms such as OpenSim, NEURON, Brian, MOtoNMS, and emerging frameworks like NEUROiD and NfMBS enable high-resolution modeling of the neuromechanical system, offering a bridge between research and practice. Despite their potential, these tools are still underutilized in clinical settings due to technical barriers, lack of standardization, and limited accessibility for non-specialists.

To address these limitations, greater integration between clinicians and model developers is essential. Simulation tools must become more user-friendly, with simplified pipelines (e.g., MOtoNMS for motion/EMG integration), clearer documentation, and training initiatives tailored to healthcare environments. Moreover, clinical studies such as those by Romanato et al. (2022) and Gogeascoechea et al. (2020) illustrate the feasibility of using these platforms in real-world rehabilitation contexts, highlighting their value in patient stratification, treatment planning, and outcome prediction.

Computational tools can further enhance the interpretation of neurophysiological and biomechanical data. For instance, systems like ReMoTo (Cisi and Kohn, 2008) and the pooled scalogram method (Borzelli et al., 2025) allow fine-grained analysis of motor neuron recruitment and muscle co-activation, especially when linked to functional clinical metrics such as gait variability or strength asymmetry.

Looking ahead, simulation environments like NEUROiD hold the promise of creating patient-specific ‘digital twins’ that could support adaptive and predictive rehabilitation planning. Nevertheless, the full integration of these tools into standard care will require robust data infrastructures, interprofessional collaboration, and regulatory pathways for clinical validation. The use of CMC as a biomarker holds potential for tracking recovery and tailoring therapies. Actionable parameters such as force deviation and co-contraction indices directly relate to functional outcomes, though further validation is required to establish clinical thresholds. While CMC is key to understanding neuroplastic adaptations, pairing it with biomechanical indicators such as movement smoothness or torque balance can provide a more holistic view of motor recovery. In the study of motor control, functional connectivity measures such as corticomuscular coherence (CMC), coherence strength correlation (CSC), and intermuscular coherence (IMC) provide valuable insights into the neural coordination underlying motor tasks. These metrics allow researchers to differentiate between neural pathways converging on spinal motor neurons and to identify shared neural information across spatially distributed muscles (Boonstra et al., 2015). Alongside these coherence-based approaches, muscle synergy analysis has emerged as a powerful tool to explore how the CNS simplifies motor control by activating groups of muscles in a coordinated manner. However, muscle synergies alone do not fully capture the complexity of the neural mechanisms involved in human movement. In fact, the synchronization of oscillatory activity within the neuromuscular system, quantified through coherence, reflects the functional coupling required for effective movement control (Ortega-Auriol et al., 2023). Therefore, combining muscle synergy analysis with measures of neural connectivity might be beneficial to achieving a more comprehensive understanding of motor coordination (Borzelli et al., 2024).

Furthermore, wearable technologies and machine learning could refine data collection and analysis, paving the way for personalized rehabilitation strategies. This integrated approach represents a promising step forward in advancing both the assessment and treatment of individuals with neurological impairments. However, several challenges remain. Small sample sizes limit the generalizability of findings, as recruiting participants with specific conditions is often difficult. Additionally, variations in study protocols, such as differences in EEG configurations, filtering techniques, and movement tasks, complicate cross-study comparisons. Many studies also rely on static tasks or isolated movements, which may not fully capture real-world motor behaviors. Future research should prioritize standardized protocols in terms of technologies and parameters analyzed and larger, more diverse cohorts to strengthen conclusions. Moreover, the growing interest in artificial intelligence, particularly deep learning, within neurorehabilitation holds promise for integrating MMC technologies into clinical practice. As highlighted by Lam et al. (2023), advances in MMC development enable more cost-effective motion capture solutions. These technological improvements may support broader clinical adoption and facilitate future research on movement patterns and motor function in individuals with neurological impairments.

Ultimately, the shift from isolated experimental protocols to clinically integrated neurobiomechanical assessments will depend on the creation of collaborative ecosystems. These must link clinicians, researchers, engineers, and data scientists in co-designing solutions that are not only effective and accurate but also feasible and interpretable in the clinical environment.

## 8 Conclusion

In this review, we examined the technologies and extracted parameters used for neurobiomechanical assessments in the context of neurological disorders. Although the selection of neurophysiological and biomechanical parameters is relatively consistent across the collected studies, substantial variations in motor tasks and experimental setups make replication challenging. To address this limitation, a key objective for future research should be the development of standardized guidelines. Such guidelines would assist researchers and clinicians in navigating the complexity of human movement in neurological populations, thereby improving reproducibility and enhancing cross-study comparability.

Although clinical scales remain widely used in rehabilitation settings, technologies that objectively quantify motor performance (e.g., MoCap systems, WS, and force platforms) provide additional insights often missed by conventional clinical assessments. Furthermore, a multidomain assessment based on a neurobiomechanical approach allows for the simultaneous evaluation of both neurophysiological and biomechanical aspects of movement. This could provide new insights not only for diagnosis but also for early intervention and preventive care. In conclusion, neurobiomechanics could offer a powerful approach to capture the complexity of motor impairment. By combining experimental approaches with computational modeling and clinical insight, this field moves closer to enabling precision rehabilitation. Despite these promising preliminary findings, the multidomain approach remains largely confined to research environments. Its clinical adoption is limited by several challenges, including the high cost of technological equipment and the need for specialized personnel, not only to set up the appropriate instrumentation for each patient but also to analyze and interpret the resulting data.

## Footnotes

^1^ https://opensim.stanford.edu/

^2^ http://www.neuron.yale.edu
